# Neuroimaging findings in children with COVID-19 infection: a systematic review and meta-analysis

**DOI:** 10.1038/s41598-024-55597-2

**Published:** 2024-02-27

**Authors:** Ghida Hasan Safadieh, Rania El Majzoub, Linda Abou Abbas

**Affiliations:** 1 Neuroscience Research Center, Faculty of Medical Sciences, Lebanese University, Hadath, 1003 Lebanon; 2 School of Pharmacy (Department of Biomedical Sciences), Lebanese International University, Mazraa, 146404 Lebanon; 3 Neuroscience Research Center, Faculty of Medical Sciences, Lebanese University, Beirut, Lebanon

**Keywords:** Health care, Medical research, Neurology

## Abstract

The COVID-19 pandemic has impacted individuals differently, and there's been a growing body of evidence pointing to neurological complications caused by the virus. However, our understanding of the range of neurological issues linked to SARS-CoV-2 infection in children is limited. This systematic review and meta-analysis aimed to assess the abnormal neuroimaging findings in pediatric COVID-19 patients, shedding light on this crucial aspect of the disease's impact on children. We conducted an extensive search in the PubMed, Medline, and ScienceDirect databases for observational studies reporting neuroimaging findings of the brain and spinal cord in children with COVID-19 between December 1, 2019, and October 30, 2021. Grey literature sources, including medRxiv and Google Scholar, were also explored. Pooled proportions of abnormal neuroimaging findings, categorized into neurovascular findings, ADEM-like lesions, encephalitic pattern, myelitis, transient splenial lesions, and other anomalies, were calculated using a random-effects model. Between-study heterogeneity was assessed using the χ^2^ statistic for pooled proportions and the inconsistency index *I*^2^. The Quality of the studies was evaluated using the NIH Quality Assessment Tool and the adapted Newcastle–Ottawa Scale. Our search yielded 9,605 articles, with 96 studies (involving 327 pediatric patients) included in the qualitative analysis. Of these, five reports (encompassing 111 patients) underwent quantitative analysis. The pooled proportion of pediatric COVID-19 patients with neurological symptoms and exhibiting abnormal neuroimaging findings was 43.74%. These findings were further categorized into neurovascular findings (8.22%), ADEM-like lesions (7.69%), encephalitic pattern (13.95%), myelitis (4.60%), transient splenial lesions (16.26%), and other abnormalities (12.03%). Insignificant between-study heterogeneity was observed in all categories, and our analysis did not reveal significant publication bias. In conclusion, a substantial proportion of pediatric COVID-19 patients with neurological symptoms have abnormal neuroimaging findings, underscoring the need for vigilant monitoring of neurological complications in this vulnerable population. Standardized reporting and long-term follow-up studies are essential to fully understand the implications of these findings. Collaborative research efforts will deepen our understanding of COVID-19's neurological dimensions in children and enhance clinical care for this population.

## Introduction

The coronavirus disease 2019 (COVID-19) pandemic, stemming from the highly pathogenic severe acute respiratory syndrome coronavirus 2 (SARS-CoV-2), emerged in December 2019 and swiftly evolved into a global crisis, affecting millions of individuals worldwide. With over 600 million confirmed cases and more than 6 million confirmed deaths as of March 2023^1,2^, the pandemic has underscored the urgent need for a comprehensive understanding of its multifaceted impacts on human health.

Initially characterized by predominantly respiratory symptoms, COVID-19's complex clinical presentation has expanded to encompass a diverse array of manifestations. While the elderly with coexisting health conditions bore the brunt of severe outcomes, the pediatric population experienced comparatively mild disease courses, with some children remaining asymptomatic altogether^3^. However, as the pandemic progressed, the constellation of symptoms associated with COVID-19 extended beyond respiratory involvement, increasingly encompassing neurological manifestations^4^.

The neurological facet of COVID-19 has garnered significant attention due to its potential implications for both acute clinical care and long-term health outcomes. Although non-specific neurological symptoms such as headache, anosmia, dysgeusia, dizziness, disturbed consciousness, and paresthesia have been reported, a growing number of adult COVID-19 patients have exhibited distinct neurological conditions, including acute cerebrovascular disease, encephalitis, seizure, Guillain-Barré syndrome (GBS), and Miller Fisher syndrome (MFS)^5–7^. The surge in published literature has documented substantial incidences of abnormal neuroimaging findings in adults with COVID-19, adding complexity to the spectrum of the disease's presentation.

In the pediatric population, the scenario is equally intricate. An immune-mediated syndrome known as pediatric multisystem inflammatory syndrome, emerging during the latent phase of COVID-19, has been documented, with some children experiencing neurological symptoms accompanied by alterations in brain imaging^8^. The range of neuroimaging findings in children with COVID-19 encompasses cytotoxic lesions of the corpus callosum (CLOCCs), idiopathic intracranial hypertension (IIH), arterial ischemic stroke (AIS), hemorrhagic posterior reversible encephalopathy syndrome (PRES), and acute disseminated encephalomyelitis (ADEM)^9–13^. Notably, the burgeoning literature on COVID-19-related neuroimaging findings in children has largely consisted of case reports and series, which, while informative, possess limitations in providing comprehensive evidence-based incidence data.

Addressing this research gap, the current study embarks on a systematic review and meta-analysis to collate the diverse radiological findings reported in the evolving corpus of literature on children with COVID-19. By employing rigorous methodology, this study aims to synthesize data from various observational study designs. Ultimately, our objective is to derive a comprehensive and pooled estimate of the incidence of neuroimaging abnormalities in this pediatric population. This endeavor not only contributes to enhancing our understanding of the neurological aspects of COVID-19 in children but also provides vital evidence for radiologists and clinicians faced with interpreting neuroimaging findings in the context of COVID-19.

## Methods

This systematic review and meta-analysis were conducted following the Preferred Reporting Items for Systematic Reviews and Meta-Analyses (PRISMA) guidelines^14^.

### Literature search

A comprehensive search was executed in PubMed, Medline, and ScienceDirect databases to identify studies published between December 1, 2019, and October 30, 2021. For PubMed and Medline, the search utilized MeSH terms and keywords in the title/abstract field: (Coronavirus disease OR Novel coronavirus OR 2019-nCoV OR SARS-CoV-2 OR Covid-19 OR Severe Acute Respiratory Syndrome Coronavirus 2) AND (Brain OR Central nervous system OR CNS OR spinal cord OR peripheral nervous system OR PNS OR neurological) AND (CT OR computed tomography OR MRI OR magnetic resonance imaging OR neuroimaging OR imaging OR neuroradiology OR radiology) AND (Children OR child OR pediatric OR "child, preschool"[MeSH Terms] OR "Adolescent"[MeSH Terms]). This search was conducted on April 16, 2022.

In ScienceDirect, due to limitations in allowed Boolean terms, three separate searches were conducted for relevant imaging-related keywords and COVID-19, with the final results merged. This search was performed on April 17, 2022. The following keywords, with the title/abstract/keywords field and a filter restricting results to 2019/2020/2021, were used: (CT OR computed tomography OR MRI OR magnetic resonance imaging OR imaging OR neuroimaging OR radiology) AND (Child OR pediatric), (Coronavirus disease OR Novel coronavirus OR 2019-nCoV OR SARS-CoV-2 OR Covid-19 OR Severe Acute Respiratory Syndrome Coronavirus 2) AND (child OR pediatric), (Brain OR Central nervous system OR CNS OR spinal cord OR peripheral nervous system OR PNS OR neurological) AND (child OR pediatric).

Additional sources were identified by reviewing reference lists of relevant articles. Grey literature was explored through platforms like medRxiv using the following keywords in full text, abstract, or title: "Coronavirus disease" AND neurological AND imaging AND Children, with a filter for 2019 to 2021. Google Scholar was also used with the same keywords as PubMed and Medline, and filters set for anywhere in the article and 2019 to 2021. Manual searches of related articles were also conducted. These websites were accessed on 4/1/2023, 2/2/2023, and 28/2/2023, respectively. The literature search was conducted by a single reviewer. The author was not blinded to authors, institutions, or journals during study selection or data extraction. Literature management was facilitated using EndNote version X9^15^.

### Inclusion and exclusion criteria

Included in this study were investigations of imaging findings of the brain and spinal cord in children with COVID-19. The following inclusion criteria were applied: (1) Population: Children (age ≤ 18) diagnosed with COVID-19. (2) Study design: All observational studies (case-series, case reports, cross-sectional, case–control, and cohort studies) were eligible. (3) Studies conducted between December 2019 and October 2021. (4) Outcomes: Imaging findings of the brain and spinal cord in COVID-19 patients with neurological symptoms. Only imaging findings at presentation were considered, excluding findings after treatment or follow-up.

Exclusion criteria were as follows: (1) Reviews, editorials, and letters. (2) Articles not written in English. (3) Non-human studies. A single reviewer reviewed the literature.

### Data extraction

Data from selected articles were extracted into standardized formats, encompassing: (a) Study characteristics: First author's name and year of publication, country of origin, study design, sample size, article quality; (b) Patient demographic and clinical details: age, sex, imaging modality and specifications, number of neuroradiologist reviewers and their experience, reported neurological symptoms, frequency and proportion of positive neuroimaging findings, specific neuroimaging findings, pre-existing medical conditions. Data extraction was performed by one reviewer and validated for accuracy.

### Quality assessment

Quality assessment of included studies was conducted by one reviewer at the study level. The NIH quality assessment tool for case reports and case series and the adapted Newcastle–Ottawa Scale for other designs were utilized for quality evaluation^16,17^.

### Synthesis methods

The synthesis of results in this systematic review employed a narrative and quantitative approach to address the research questions. Case reports, case studies, case–control, and cross-sectional studies underwent a narrative synthesis, which entailed summarizing their findings to identify diverse radiological observations. Meanwhile, cohort studies with sample sizes greater than 10 underwent quantitative synthesis, utilizing meta-analysis techniques to estimate the incidence of neuroimaging abnormalities in the pediatric population. The primary outcomes of this meta-analysis were pooled proportion estimates of abnormal neuroimaging findings, categorized as neurovascular findings (arterial or venous stroke), ADEM-like lesions (autoimmune), encephalitic pattern (Acute Hemorrhagic Necrotizing Encephalitis), myelitis (Longitudinally Extensive Transverse Myelitis), transient splenial lesions (RESLES, MERS), and others (PRES, neuritis, brain edema, etc.).

For meta-analytic pooling of data, the random-effects model with variance stabilization was used, employing the Freeman-Tukey double arc-sine transformation^18^. Pooled proportions with 95% confidence intervals were derived using the Der Simonian-Laird random-effects model^19^. Between-study heterogeneity was assessed using χ^2^ statistics for pooled estimates (P < 0.05 indicating significant heterogeneity) and the Higgin's inconsistency index (I^2^), where I^2^ values of 0–40%, 30–60%, 50–90%, and 75–100% indicated insignificant, moderate, substantial, and considerable heterogeneity, respectively^20^. Publication bias was evaluated through Funnel plots, the Beggar's test, and Egger's tests^21^. All statistical analyses were performed using R^22^. A P-value of < 0.05 was considered statistically significant.

## Results

**Figure 1:**
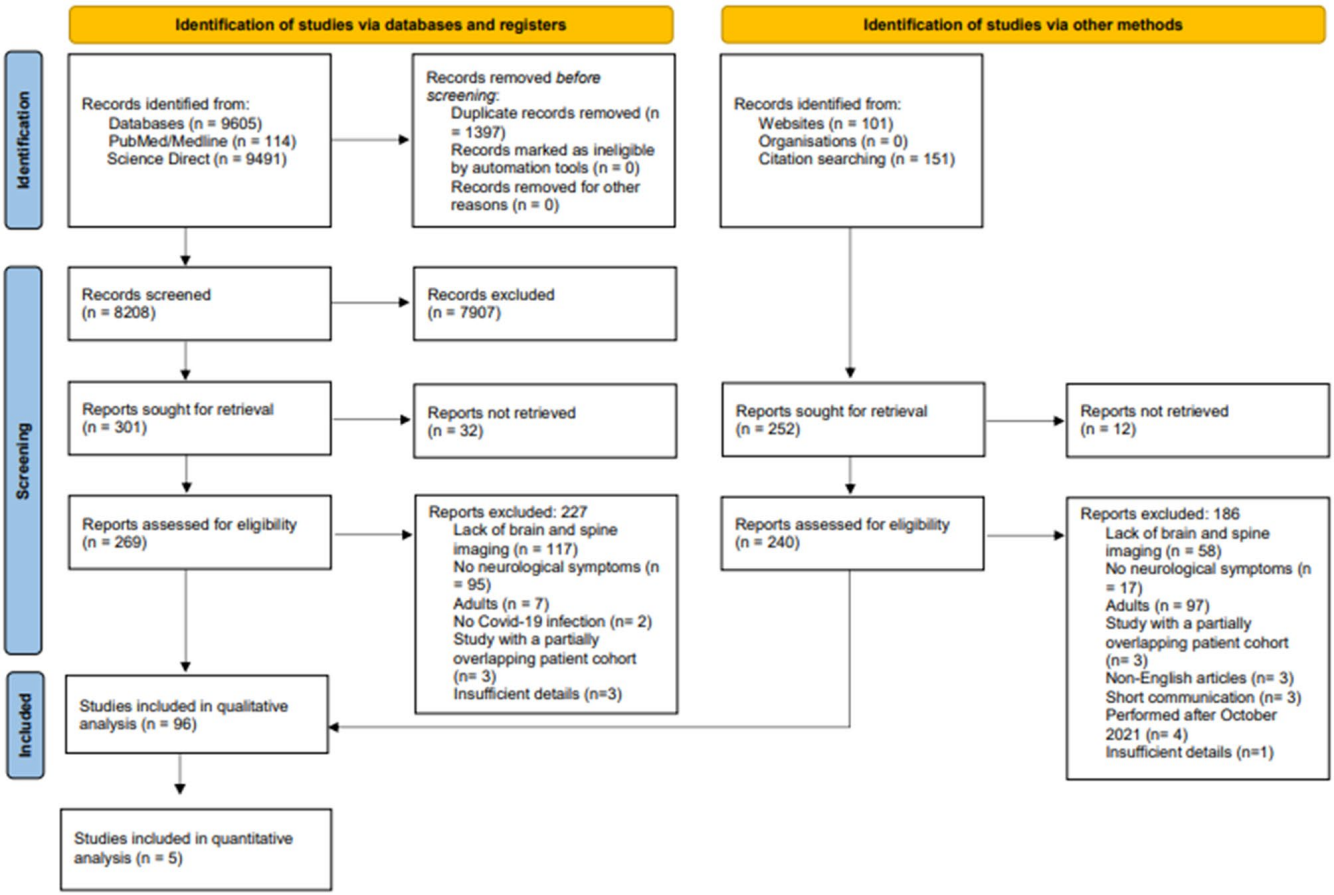
Flow diagram depicting the study eligibility criteria.

A flowchart representing the publication selection process is presented in Fig. 1. The initial literature search yielded 9605 articles. After removing duplicates, 8208 articles were subjected to eligibility screening. Among these, 7907 were excluded based on the assessment of their titles and abstracts, and 32 reports were not accessible. The full texts of the remaining 269 articles were comprehensively reviewed; 227 articles were further excluded due to various reasons such as lack of brain and spine imaging, lack of neurological symptoms, partial overlap with patient cohorts, inclusion of adult populations, insufficient details, and absence of Covid-19 infection. In cases of overlapping cohorts, preference was given to the study with more relevant information about abnormal findings. An additional 252 studies were identified through other methods (reference lists, Google Scholar, medRxiv), out of which 240 were thoroughly reviewed for eligibility. Among these, 186 were excluded due to similar reasons as mentioned before. In total, 96 articles, involving a cumulative 327 patients, were included in the qualitative review. Within the 96 articles, 5 reports encompassing 111 patients were considered for quantitative analyses^23–29^.

The characteristics of the 96 included studies are summarized in Table 1 and Supplementary Tables S1, S2 and S3. Among these, 75 were categorized as case reports or case series, while 21 were classified as cross-sectional, case–control, or cohort studies. Within the latter group, 17 studies were retrospective, 3 were prospective, and one exhibited both retrospective and prospective elements. The selected studies originated from diverse countries, including China, Turkey, Iran, Argentina, Bangladesh, Brazil, Chile, France, India, Ireland, Italy, Kosovo, Saudi Arabia, Mexico, Peru, Republic of Macedonia, Spain, Switzerland, the UK, and the USA. One study was a multinational collaboration involving France, the UK, the USA, Brazil, Argentina, India, Peru, and Saudi Arabia. Among the included studies, 49 employed MR as the sole neuroimaging modality, 10 utilized CT exclusively, and 35 employed various modalities like MR, CT, US, and OCT. Two studies did not report the modalities used^30,31^.

**Table 1:** The characteristics of the included studies (cross-sectional, case–control, and cohort studies).

First author's name	Year of publication	Country of origin	Study design	Sample size	Imaging modality (n)	Number and proportion of patients with positive imaging findings
Emami	2020	Iran	Cross-sectional study	2	CT (1), MRI (1), US (1)	1(1/2)
Kushwaha	2020	India	Cross-sectional study	1	MRI	0
Coronado Munoz	2021	Peru	Prospective case–control study	11	MRI (1), CT (10)	11(11/11)
Yan	2021	China	Prospective case–control study	5	MRI (5)	4(4/5)
Ray	2021	UK	Prospective cohort study	42	MRI (38), CT (10), MRA (1)	25(25/42)
Balagurunathan	2021	India	Retrospective and prospective cohort study	3	NR	0
Aksu Uzunhan	2021	Turkey	Retrospective cohort study	2	MRI (2)	2(2/2)
Biko	2021	USA	Retrospective cohort study	8	MRI (7), CT (6)	3(3/8)
Caro-Domínguez	2021	Spain	Retrospective cohort study	12	MRI (9), CT (3)	2(2/12)
Elmas	2021	Turkey	Retrospective cohort study	7	MRI (7)	1(1/7)
Fenlon Iii	2021	USA	Retrospective cohort study	4	MRI (4)	2(2/4)
Gupta Dch	2021	India	Retrospective cohort study	9	CT (8), USG (1)	3(3/9)
Olivotto	2021	Italy	Retrospective cohort study	4	MRI (4)	1(1/4)
Orman	2021	USA	Retrospective cohort study	20	CT (17), MRI (17),MRV (2), and MRA (7)	2(2/20)
Oualha	2020	France	Retrospective cohort study	1	MRI	1(1/1)
Palabiyik	2021	Turkey	Retrospective cohort study	21	MRI (21)	10(10/21)
Paterson	2020	UK	Retrospective cohort study	2	MRI (2)	2(2/2)
Penner	2021	UK	Retrospective cohort study	16	MRI (16)	7(7/16)
Riollano-Cruz	2021	USA	Retrospective cohort study	1	CT	1(1/1)
Salman	2021	Turkey	Retrospective cohort study	1	MRI	1(1/1)
Ucan	2021	Turkey	Retrospective cohort study	3	MRI (3), CT (1)	1(1/3)

*NR* not reported.

Table 2 and Supplementary Table S4 provide detailed information about the neuroimaging findings in children with COVID-19. Supplementary Table S4 contains detailed information on neuroimaging findings in case reports and case series studies, while Table 2 provides detailed information on neuroimaging findings in other types of observational studies. For cross-sectional and case–control studies with low sample sizes, only descriptive analysis was performed. In contrast, meta-analysis was conducted for cohort studies with a sample size greater than 10.

**Table 2:** Detailed neuroimaging findings in children with COVID-19 (cross-sectional, case–control, and cohort studies).

First author's name	Neuroimaging findings
Aksu Uzunhan	Splenial diffusion restriction (2/2)
Balagurunathan	Normal
Biko	Sinus thrombosis (1/8); cerebral edema (1/8); posterior cerebral stroke (1/8)
Caro-Domínguez	Leptomeningeal enhancement of the right precentral sulcus (1/12); foci of restricted diffusion in the splenium of the corpus callosum (1/12)
Coronado Munoz	Diffuse brain edema (1/11); Diffuse brain edema, hypodense cortical and sub-cortical right lesion, r/o blood, interhemispheric and tentorial area meningeal enhancement (1/11); Basal ganglia hematoma, right ventricle hemorrhage, midline leftward shift, brain edema, brain herniation (1/11); Diffuse brain edema, subcortical hypodensities (1/11); Loss of white – gray matter differentiation (1/11); Hypoxic ischemic changes, occipital subarachnoid hemorrhage (1/11); Hemorrhagic lesion in right frontotemporal region, midline shift, brain herniation (1/11); Multifocal hyperintense white matter lesions in frontotemporal region (1/11); Ischemic lesion in left basal ganglia, Diffuse cortico-subcortical atrophy (1/11); Diffuse edema, parietal laminar subdural hematoma (1/11); intraventricular hemorrhage left lateral and posterior with acute hydrocephalus (1/11)
Elmas	Diffusion restriction in corpus callosum splenium (1/7)
Emami	Right occipital mass and intracerebral hemorrhage (1/2)
Fenlon Iii	Hyperintensity/ restricted diffusion involving the bilateral parieto-occipitalcortices with mild cortical thickening, and punctate T2/FLAIR hyperintensity in the left frontoparietal centrum semiovale (1/4), papilledema (1/4)
Gupta Dch	Subacute infarct in right parietooccipital region and left thalamus (1/3); Acute to subacute infarct in right middle cerebral artery territory (1/3); Oedema left frontotemporal region (1/3)
Kushwaha	Normal
Olivotto	Enhancement of the anterior roots of the cauda equina (1/4)
Orman	T2-FLAIR hyperintensity and cortical edema in the occipital lobes, consistent with posterior reversible encephalopathy syndrome (1/20); subtle right hippocampal T2-FLAIR signal alteration with corresponding edema (1/20)
Oualha	Sphenoidal sinusitis with cavernous sinus thrombosis
Palabiyik	Diffusion restriction was detected in the posterior part of the splenium in diffusion-weighted MRI sequences (6/21); symmetrical signal changes in the cerebellar hemispheres, periaqueductal region, mesencephalon, bilateral hypothalamic region, bilateral thalamus, lentiform nucleus, caudate nucleus, deep white matter, and subcortical area with no diffusion restriction or contrast enhancement, new pathological signal changes were in the bilateral parietooccipital and bilateral frontoparietal regions without contrast enhancement or diffusion restriction (1/21); in the bilateral frontoparietal region, and bilateral parietal lesions with cortiko-subcortical symmetrical diffusion restriction and contrast enhancement were detected, laminar necrosis (1/21); diffuse contrast involvement in the cauda equina fibers and nerve roots (1/21); cerebral and cerebellar atrophy, as well as bilateral symmetrical diffuse signal changes and volume loss in periventricular deep white matter (1/21)
Paterson	Dilated optic nerve sheaths and narrowed but patent trans verse sinuses; consistent with raised intra-cranial pressure (1/2); Extensive, symmetrical bilateral signal changes without restricted diffusion involving the cortices of the cerebral and cerebellar hemispheres and the thalami (1/2)
Penner	Splenial signal changes (4/16), micro haemorrhages (3/16), subcortical parietal white matter lesions (3/16), leptomeningeal myopathic and enhance ment (1/16), and cerebral oedema (1/16)
Ray	D44 cortical & basal ganglia T2 lesions & subcortical white matter lesions consistent with ADEM (1/42); multiple lesions T2/FLAIR signal change within the deep/juxtacortical & subcortical white matter, brainstem; abnormal signal thoracic spine extending to conus (1/42); Multiple T2/flair lesions involving the thalami, internal capsules, basal ganglia, parietal white matter, R middle cerebellar peduncle & cerebellar white matter (1/42); T2 diffuse hyperintensity of the cord from C1 to conus.Brain T2 diffuse hyperintensities bilaterally (1/42); Multiple inflammatory WM lesions in juxtacortical, periventricular, pericallosal & infratentorial, lesions demonstrated 'open ring' contrast enhancement & restricted diffusion (1/42); signal change in intra orbital segment of R optic nerve consistent with optic neuritis; Syrinx (incidental) (1/42); enhancement of the spinal nerves throughout with florid enhancement of the cauda equina, suggestive of GBS (1/42); spine not tolerated (1/42); enhancement of the cranial nerves & cauda equina nerve roots (1/42); T2 signal abnormality involving the hippocampi (not atrophic). Cortical diffusion restriction (1/42); MERS (2/42); no acute changes. Established L anterior basal ganglia stroke (1/42); Acute R anterior & middle cerebral artery territory infarction (1/42); Large intraparenchymal haemorrhage R frontal lobe with midline shift (1/42); T2 Hyperintensity seen within the claustra bilaterally, generalised parenchymal volume loss (1/42); Developmental venous anomaly (incidental finding) (1/42); Isolated area of leptomeningeal inflammation L post-parietal lobe (1/42); Bi-frontal multiple focal areas of diffusion restriction (1/42); diffuse microhaemorrhages (1/42); Diffuse cortical signal abnormality both cerebral hemispheres (1/42); MERS & multi-focal hazy signal change of WM (no diffusion restriction) (1/42); MERS & cortical/subcortical diffusion restriction L frontal & R occipital lobes (1/42); Abnormal cortical T2 signal in the occipito-parietal regions (L > R) consistent with PRES. Mild diffusion restriction of the L hippocampus & thalamus (seizure-associated) (1/42); Small non-specific T2/FLAIR hyperintensities bi-parietal subcortical white matter (1/42)
Riollano-Cruz	Near total right middle cerebral artery infarction involving cortex, subcortical white matter and deep gray matter, left frontal subarachnoid hemorrhage
Salman	Reversible lesion was found in the corpus callosum splenium
Ucan	Focal area of hyperintensity in the splenium of the corpus callosum corresponding to a focal area of restricted diffusion on diffusion- weighted and apparent diffusion coefficient images (MERS) (1/3)
Yan	Hypoxic changes, signal changes of varying intensity in the basal ganglia region in T1WI (2/5); brain hypoplasia with delayed myelination (2/5)

**Figure 2:**
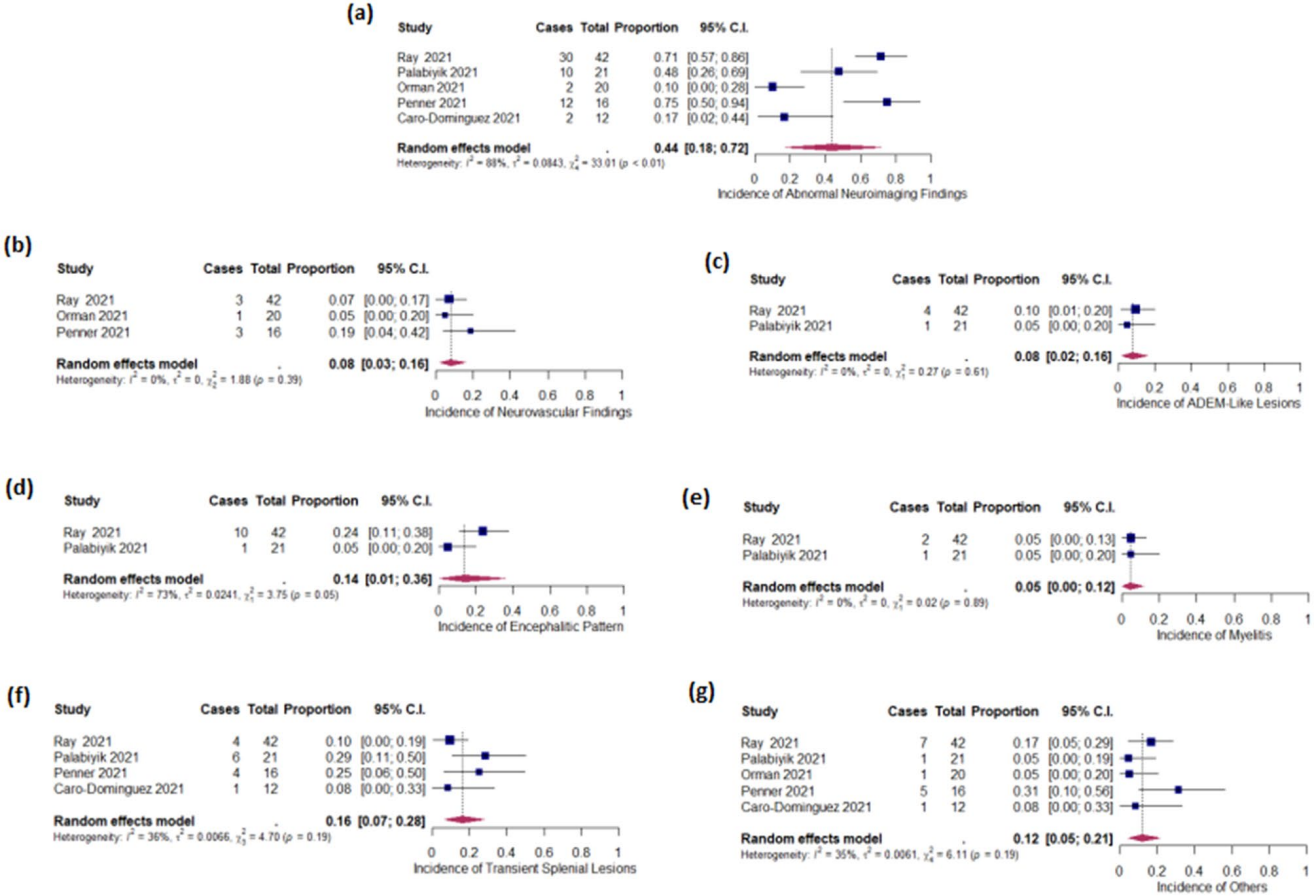
Forest plots of pooled proportions. Forest plots of pooled proportions of (**a**) Abnormal neuroimaging findings, (**b**) Neurovascular findings, (**c**) ADEM-like lesions, (**d**) Encephalitic pattern, (**e**) Myelitis, (**f**) Transient splenial lesions, and (**g**) Other events in children with COVID-19.

In the pool of included studies, 43.74% (95% CI 17.55 to 71.77%) of the children exhibited abnormal neuroimaging findings. Please refer to Supplementary Table S5 for detailed information. The pooled incidence of neurovascular findings was 8.22% (95% CI 2.57 to 16.00%; I^2^ = 0.00%). The pooled incidence of ADEM-like lesions was 7.69% (95% CI 1.89 to 16.13%; I^2^ = 0.00%). The pooled incidence of encephalitic pattern was 13.95% (95% CI 0.86 to 36.15%; I^2^ = 73.32%). The pooled incidence of myelitis was 4.60% (95% CI 0.33 to 11.87%; I^2^ = 0.00%). The pooled incidence of transient splenial lesions was 16.26% (95% CI 6.93 to 28.02%; I^2^ = 36.19%). The pooled incidence of other abnormalities was 12.03% (95% CI 4.75 to 21.48%; I^2^ = 34.50%) (Table 3 and Fig. 2).

**Table 3:** Summary of the meta-analytically pooled proportions.

	Summary estimate		
	Pooled incidences (%) [95% CI]	P-value for heterogeneity^a^	I^2b^ (%)
Neurovascular findings	8.22 [2.57–16.00]	0.3899	0.00
ADEM-like lesions	7.69 [1.89–16.13]	0.6053	0.00
Encephalitic pattern	13.95 [0.86–36.15]	0.0529	73.32
Myelitis	4.60 [0.33–11.87]	0.8905	0.00
Transient splenial lesions	16.26 [6.93–28.02]	0.1950	36.19
Others	12.03 [4.75–21.48]	0.1913	34.50

*CI* confidence interval. I^2^ = Higgins’ inconsistency index.

^a^P-value of the χ^2^ statistics to test the heterogeneity of the pooled data (P < 0.05 indicates significant heterogeneity).

^b^Higgins’ inconsistency index (0–40% may indicate insignificant heterogeneity; 30–60%, 50–90%, and 75–100% may indicate insignificant, moderate, substantial, and considerable heterogeneity, respectively).

**Figure 3:**
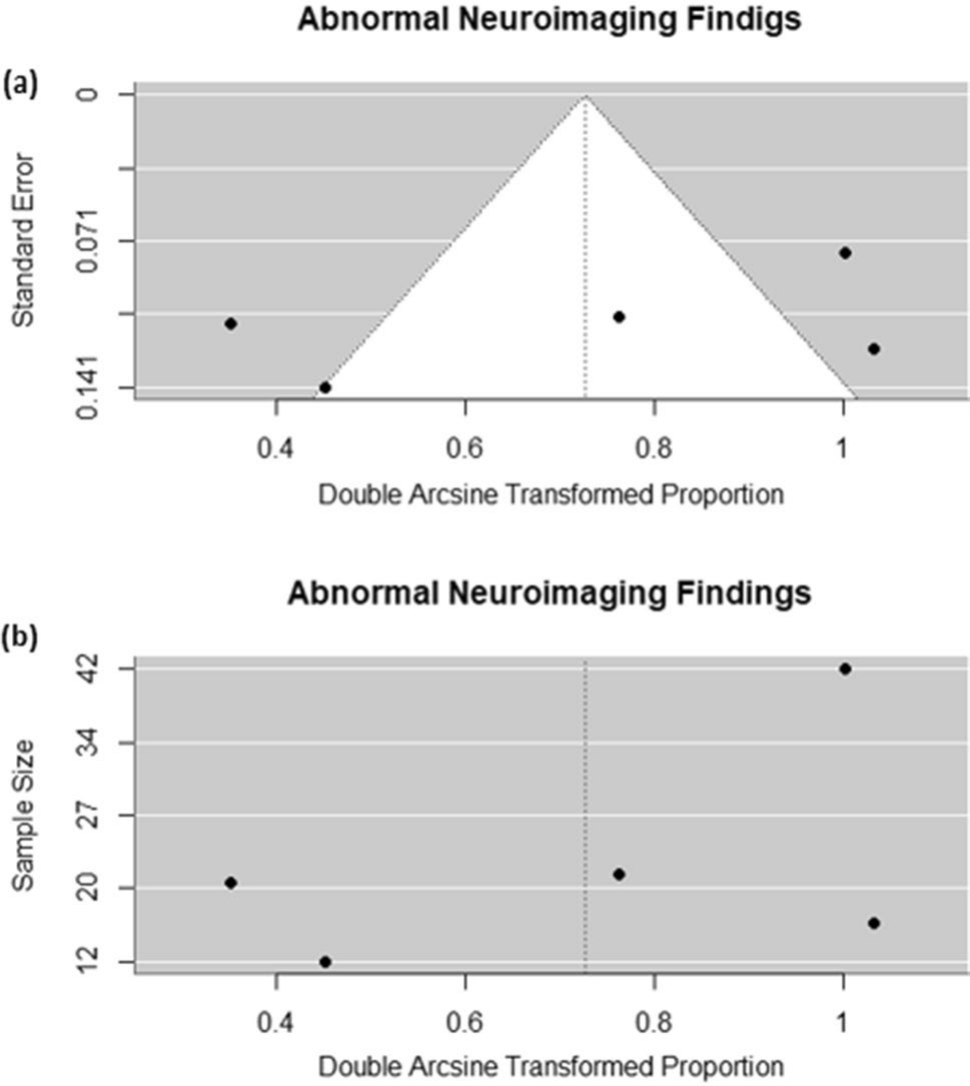
Funnel plots of pooled proportions. Funnel plots of pooled proportions of (**a**) & (**b**) Abnormal neuroimaging findings in children with COVID-19.

All included studies exhibited no significant publication bias in funnel plots (indicating symmetric distribution of studies), Beggar’s test (P = 0.4833), and Egger’s tests (P = 0.3369) (P > 0.05) (Fig. 3). All included studies had insignificant between-study heterogeneities (P > 0.05) (Table 3).

**Figure 4:**
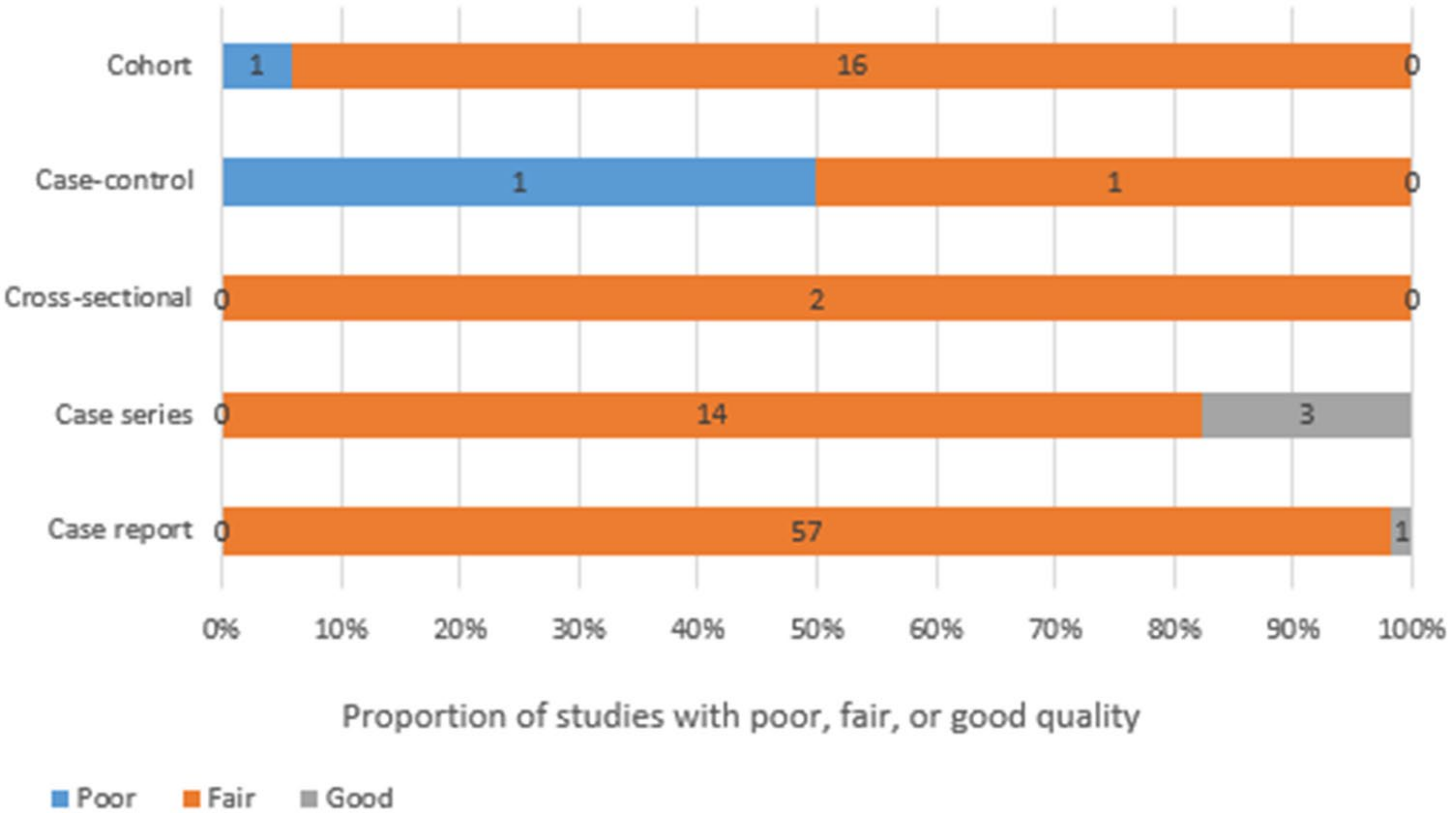
The quality assessment of included studies. Proportion of studies with poor, fair, or good quality categorized by study type.

The quality assessment, utilizing the NIH quality assessment tool for case report and case series studies, and the Newcastle–Ottawa Scale (NOS) for other study designs, indicated an overall fair quality, categorized as good, fair, or poor on the quality scale (Fig. 4).

## Discussion

The emergence of the COVID-19 pandemic caused by the novel coronavirus, SARS-CoV-2, has evolved into a global health crisis. Initially focused on respiratory symptoms, it's now clear that COVID-19 presents a complex clinical picture affecting various organ systems^32,33^. Neurological manifestations have gained attention for their potential impact on both immediate clinical care and long-term health outcomes^34,35^. This systematic review and meta-analysis aimed to provide a comprehensive assessment of the abnormal neuroimaging findings in children with COVID-19.

Our findings reveal that a substantial proportion of pediatric COVID-19 patients with neurological symptoms exhibit abnormal neuroimaging findings, with 43.74% of children in the included studies demonstrating such abnormalities. These findings underscore the importance of considering neurological complications in the management of pediatric COVID-19 cases.

In a systematic review conducted by Choi et al. an extensive exploration was undertaken to comprehensively assess the spectrum of COVID-19-related neurological manifestations and associated abnormal neuroimaging findings in adults. Their meta-analysis unveiled that 42.6% of adult patients exhibited abnormal neuroimaging findings, as observed through brain CT or MRI^36^. With acute to subacute infarcts were the most common (24.0%), followed by cerebral micro hemorrhages (6.9%), acute spontaneous intracerebral hemorrhages (5.4%), and encephalitis/encephalopathy (3.3%)^36^. It is important to note that their investigation exclusively centered on specific COVID-19-related neuroimaging findings in the adult population and specifically considered MRI and CT as the imaging modalities of interest, although acknowledging that alternate modalities such as OCT, and TCD are also utilized.

In contrast, our study takes a unique approach, focusing exclusively on the pediatric group of COVID-19 patients, which is an area with limited research. Additionally, we aimed to cover a wide range of neuroimaging findings using diverse range of methods commonly used in pediatric cases. This broader scope is in response to the frequent use of various imaging techniques in pediatric clinical settings, extending the depth of our investigation.

In terms of specific neuroimaging abnormalities, our analysis categorized them into neurovascular findings, ADEM-like lesions, encephalitic pattern, myelitis, transient splenial lesions, and other findings. Neurovascular findings, encompassing findings like arterial or venous stroke, exhibited a pooled incidence of 8.22%. ADEM-like lesions showed a pooled incidence of 7.69%. Encephalitic pattern, including findings like Acute Hemorrhagic Necrotizing Encephalitis, showed a pooled incidence of 13.95%. Myelitis had a pooled incidence of 4.60%. Transient splenial lesions, encompassing findings like RESLES and MERS, exhibited a pooled incidence of 16.26%. Other abnormalities, such as PRES, neuritis, and brain edema, had a pooled incidence of 12.03%. These findings emphasize the diversity of neurological involvement in pediatric COVID-19 cases, spanning from inflammatory processes to vascular events.

In a recent systematic review led by Falsaperla et al. in 2023, undertook an exhaustive examination of COVID-19-related neurological manifestations in the pediatric population. Although this study primarily emphasized clinical symptoms, it did not include a meta-analytic assessment. Nevertheless, their conclusions align closely with our own findings, albeit with distinct categorization. Their results highlighted that encephalitis represented the most frequent diagnosis, accounting for 20.83% of cases, followed by seizures (10.42%), GBS (10.42%), cerebrovascular involvement (10.42%), ADEM (8.33%), and encephalopathy (8.33%). Additionally, a fraction of cases exhibited neurological signs secondary to central nervous system lesions (4.17%), and peripheral neuropathy (4.17%)^37^.

The cellular and molecular basis of SARS-CoV-2's ability to affect the nervous system is not fully understood. In light of this, several theoretical mechanisms have been posited to shed light on the acute and postacute neurological manifestations associated with COVID-19. Firstly, one hypothesis proposes that SARS-CoV-2 exhibits an affinity for infecting olfactory neurons, subsequently spreading through axons and across the synapse, thereby causing central nervous system infection^38^. Secondly, considering the viral-induced depletion of ACE-2, there is a perturbation in the renin-angiotensin system equilibrium. Consequently, a prothrombotic state may ensue, impairing both large vessel and microvascular blood flow. This vascular dysfunction heightens the risk of thrombotic and hemorrhagic stroke following SARS-CoV-2 infection^39^. Thirdly, perhaps the most pivotal mechanism at play is immune dysregulation, culminating in autoimmunity and hyperinflammatory responses. The aberrant immune response is recognized as a central contributor to neurological involvement in COVID-19 cases^40^. Lastly, the cytokine storm and systemic hyperinflammatory responses incited by the virus present an additional avenue through which neurological manifestations may occur. Elevated pro-inflammatory cytokine levels can disrupt normal neuronal function, impede neurotransmitter systems, and induce neuronal damage^41,42^. Understanding the mechanisms responsible for the neurological manifestations of COVID-19 is crucial for the development of potential therapeutic interventions. These mechanisms can operate independently or in concert within individual patients, giving rise to a range of clinical and neuroimaging presentations that, while varied, often exhibit overlapping features. This underscores the significance of investigating these mechanisms in our pursuit of effective treatments^43^.

The observed incidence of neurological abnormalities in pediatric COVID-19 patients raises several important clinical and research implications. First, it highlights the necessity for a high index of suspicion for neurological complications in children with COVID-19, especially those presenting with neurological symptoms. Timely neuroimaging assessments and consultations with pediatric neurologists may be essential for early diagnosis and appropriate management.

Second, the findings of this study emphasize the importance of continued research into the long-term consequences of COVID-19 in children. Even in cases where acute neurological symptoms resolve, it remains to be seen whether these children may be at risk of long-term cognitive or neurological sequelae. Longitudinal studies are needed to assess the evolution of these abnormalities and their potential impact on a child's development and quality of life.

To the best of our knowledge, this study represents the first systematic review and meta-analysis of neuroimaging findings in pediatric SARS-CoV-2 cases. Nonetheless, it is important to acknowledge several limitations of this study. Firstly, some of the studies included in our analysis exhibited substantial heterogeneity, attributed to variations in study designs, patient cohorts, and imaging protocols. This inherent diversity, though mitigated through statistical methods, somewhat limits the generalizability of our findings. Secondly, the potential presence of publication bias, although not overtly evident in our assessments, cannot be entirely dismissed. Thirdly, the relatively limited number of studies constrained our ability to conduct more detailed subgroup analyses and explore specific aspects comprehensively. Moreover, in this study, our research commenced on December 1, 2019. It is essential to acknowledge a limitation related to the evolving nature of information about COVID-19 during that early period. Detailed and comprehensive information about COVID-19, including its clinical manifestations and neurological implications, may not have been readily available at the beginning of our study. As a result, some aspects of our analysis may be based on limited data and evolving knowledge in the early stages of the pandemic. Furthermore, establishing a causal relationship between COVID-19 infection and the observed neuroimaging abnormalities remains an ongoing area of investigation. These abnormalities may be influenced by systematic confounding factors such as comorbidities, mechanical ventilation, and the complex pharmacological regimens administered for respiratory distress with hypoxia. These limitations underscore the necessity for future prospective studies that consider comorbidities and conduct more intricate analyses to affirm the potential association between COVID-19 and neuroimaging findings.

## Conclusion

In conclusion, this systematic review and meta-analysis highlight the incidence of abnormal neuroimaging findings in children with COVID-19. The findings underscore the importance of vigilance for neurological complications in pediatric COVID-19 cases, as well as the need for standardized reporting and long-term follow-up to better understand the implications of these abnormalities. Further research and collaboration are essential to deepen our understanding of the neurological aspects of COVID-19 in children and to improve clinical care for this vulnerable population.

### Supplementary Information

Supplementary Information.

## Data Availability

All data generated or analyzed during this study are included in this published article (and its Supplementary Information files).
