# Supplementary material for: Neuroimaging findings in children with COVID-19 infection: a systematic review and meta-analysis
Source: Sci Rep. 2024 Feb 27;14:4790. doi: 10.1038/s41598-024-55597-2 (PMC10899172; doi:10.1038/s41598-024-55597-2)
Supplement: Supplementary file 1 — Supplementary Information. [file 41598_2024_55597_MOESM1_ESM.pdf]

Neuroimaging Findings in Children with COVID-19 Infection: A Systematic Review and Meta-analysis

Ghida Hasan Safadieh[1, *], Rania El Majzoub[2], Linda Abou Abbas[3]

[1] Neuroscience Research Center, Faculty of Medical Sciences, Lebanese University, Hadath, 1003, Lebanon

[2] School of Pharmacy (Department of Biomedical Sciences), Lebanese International University, Mazraa, 146404, Lebanon
[*] ghida.safadieh@outlook.com
[3] Neuroscience Research Center, Faculty of Medical Sciences, Lebanese University, Beirut, Lebanon

Supplementary data

Supplementary Table 1 The characteristics of the included studies (Case-report and case-series)....1

Supplementary Table 2 Patient demographics and clinical details....3

Supplementary Table 3 Imaging Details....7

Supplementary Table 4 Detailed Neuroimaging Findings in children with COVID-19 (Case-report and case-series) 9

Supplementary Table 5 Summary of Neuroimaging Findings in Pediatric COVID-19 Studies....11

PRISMA 2020 checklist....12

Included articles....14

Supplementary Table 1. The characteristics of the included studies (Case-report and case-series)

First Author's Name	Year of Publication	Country of Origin	Study Design	Sample size	Imaging modality (n)	Number and proportion of patients with positive imaging findings
**Abel**	2020	USA	Case-report	1	CT, MRI	1 (1/1)
**Al Haboob**	2021	KSA	Case-report	1	CT, MRI, MRV, MRA	1(1/1)
**Appavu**	2021	USA	Case-report	2	MRI (2),MR VWI(2),MRA (2),CT (1)	2(2/2)
**Asif**	2020	UK	Case-report	1	CT, venogram	1(1/1)
**Bauer**	2021	USA	Case-report	2	MRI (2)	0
**Bhatta**	2020	USA	Case-report	1	CT	0
**Bhavsar**	2021	USA	Case-report	1	CT	0
**Biglari**	2021	Iran	Case-report	1	MRI	1(1/1)
**Brum**	2021	Argentina	Case-report	1	MRI	1(1/1)
**Cecchini**	2021	Italy	Case-report	1	MRI	0
**Chen**	2021	USA	Case-report	1	MRI	1(1/1)
**Curtis**	2021	USA	Case-report	1	CT, MRI	1(1/1)
**de Miranda Henriques-Souza**	2021	Brazil	Case-report	1	MRI	1(1/1)
**de Oliveira**	2021	Brazil	Case-report	1	NR	0
**Dean**	2021	USA	Case-report	1	MRI	1(1/1)
**Divya**	2021	India	Case-report	1	MRI, MRV, OCT, US	1(1/1)
**Dugue**	2020	USA	Case-report	1	MRI	0
**Farley**	2020	USA	Case-report	1	CT	0
**Foster**	2021	USA	Case-report	1	CT, MRI, MRV, MRA	1(1/1)
**Fragoso**	2021	Brazil	Case-report	1	MRI	1(1/1)
**Frank**	2021	Brazil	Case-report	1	MRI	0
**García-Howard**	2020	Spain	Case-report	1	CT, MRI	0
**Gaughan**	2021	Ireland	Case-report	1	MRI	1(1/1)
**Giannantonio**	2021	Italy	Case-report	1	MRI	0
**Gulko**	2020	USA	Case-report	1	CT, MRI, MRA, ,MR VWI, US	1(1/1)

**Hatipoglu**	2020	Turkey	Case-report	3	MRI (3)	0
**Hossain**	2020	Bangladesh	Case-report	1	MRI	0
**Hosseinpour**	2021	Iran	Case-report	1	CT, MRI	0
**Hutchison**	2020	USA	Case-report	1	MRI	0
**Insuga**	2021	Spain	Case-report	1	OCT, MRV	1(1/1)
**Jillella**	2021	USA	Case-report	1	CT, CTA, MRI	1(1/1)
**Khair**	2021	USA	Case-report	1	CT, MRI, MRV	0
**Khalifa**	2020	KSA	Case-report	1	MRI	1(1/1)
**Khera**	2021	India	Case-report	1	MRI	1(1/1)
**Khoshnood**	2021	USA	Case-report	1	MRI	1(1/1)
**Khosravi**	2021	Iran	Case-report	1	MRI, MRA, MRV	1(1/1)
**Korkmazer**	2021	Turkey	Case-report	1	MRI	1(1/1)
**Lazarte-Rantes**	2021	Peru	Case-report	1	MRI	1(1/1)
**McLendon**	2021	USA	Case-report	1	MRI	1(1/1)
**Mehra**	2020	India	Case-report	1	MRI	1(1/1)
**Powers**	2021	USA	Case-report	1	MRI	1(1/1)
**Saeed**	2020	Iran	Case-report	1	CT, MRI	1(1/1)
**Sahu**	2021	India	Case-report	1	MRI	1(1/1)
**Sarigecili**	2021	Turkey	Case-report	1	MRI	0
**Schiff**	2021	USA	Case-report	1	CT	0
**Shala**	2021	Kosovo	Case-report	1	MRI,MRA, CTA, US	1(1/1)
**Shenker**	2020	USA	Case-report	1	CT, MRI, MRA, MRV	0
**Sofijanova**	2020	Republic of Macedonia	Case-report	1	CT	1(1/1)
**Sofuoğlu**	2021	Turkey	Case-report	1	MRI, MRV	1(1/1)
**Tiwari**	2021	India	Case-report	1	CT, CTA	1(1/1)
**Tomar**	2021	India	Case-report	1	MRI	0
**Urso**	2021	Italy	Case-report	1	MRI	1(1/1)
**Vivanti**	2020	France	Case-report	1	MRI, US	1(1/1)
**Vraka**	2021	UK	Case-report	2	CT (2), MRI (2)	2(2/2)
**Wang**	2021	USA	Case-report	1	MRI	0
**Yildiz**	2021	Turkey	Case-report	1	US, MRI	1(1/1)
**Zain**	2021	USA	Case-report	1	MRI	1(1/1)
**Zubarioglu**	2021	Turkey	Case-report	1	MRI	1(1/1)
**Akçay**	2021	Turkey	Case-series	2	MRI (2), MRA (1)	2 (2/2)
**Akhondian**	2021	Iran	Case-series	5	MRI (5), CT (1)	5(5/5)
**Aljomah**	2021	KSA	Case-series	3	CT (2), angiogram (1), MRI (2)	2(2/3)
**Becker**	2021	USA	Case-series	4	CT (4), MRI (2), MRV (1), MRA (1)	2(2/4)
**Bektaş**	2021	Turkey	Case-series	1	MRI	1(1/1)
**Canham**	2020	UK	Case-series	1	CT, venography	0
**Chiotos**	2020	USA	Case-series	1	CT	1(1/1)
**Fouriki**	2020	Switzerland	Case-series	3	MRI (3)	1(1/3)
**Gaur**	2020	UK	Case-series	2	MRI (2)	2(2/2)
**Hameed**	2021	UK	Case-series	6	CT (4), MRI (3)	1(1/1)
**Khan**	2021	India	Case-series	3	MRI (2), CT (1)	3(3/3)
**Krueger**	2021	Brazil	Case-series	4	MRI (4), MRA (1), angiograph (1)	2(2/4)
**Lindan**	2021	International	Case-series	38	MRI (38), MRA (1), CT (2)	38(38/38)
**Miller**	2020	USA	Case-series	1	MRI	1(1/1)
**Ngo**	2021	USA	Case-series	4	MRI (4), MRA (2), CT (1)	2(2/4)
**Sánchez-Morales**	2021	Mexico	Case-series	6	MRI (5), CT(1)	4(4/6)
**Sandoval**	2021	Chile	Case-series	5	CT (4), MRI (2)	2(2/5)

NR = not reported

Supplementary Table. 2 Patient demographics and clinical details

First Author's Name	Age, mean ± SD	Sex, Male (%)	Reported neurological symptoms	Pre-existing medical conditions
**Abel**	2.75	1 (100%)	Somnolence, with slight facial grimace to noxious stimuli, diffuse hypotonia, and significant weakness	None
**Akçay**	9 ± 0	1 (50%)	Headache (1/2), Status epilepticus (1/2), Hallucinations (1/2), afebrile seizure (1/2), lethargy (1/2)	None
**Akhondian**	6.48 ± 4.4779	2 (40%)	Intractable convulsions (2/5), ataxia (1/5), drowsiness (1/5), low level of consciousness (1/5), weakness of lower limbs and walking disability (1/5), seizure (1/5), severe headache (1/5), neck stiffness (1/5)	NR
**Aksu Uzunhan**	13 ± 4.2426	2 (100%)	Hallucinations (1/2), disorientation (1/2), CNS dysfunction (1/2), Hyperactive delirium (1/2); Headache (1/2)	None
**Al Haboob**	11	1 (100%)	Headache, diplopia, bilateral sixth nerve palsy and double vision on his lateral gaze, evident bilateral ptosis and ataxic gait, generalized tonic-clonic seizures, ophthalmoplegia, weak gag reflex, absent cough, and absent deep tendon reflexes	None
**Aljomah**	6.3333 ± 5.5075	2 (66.66%)	Headache (2/3), Dysarthria (1/3), gait instability (1/3), double vision (1/3), ophthalmoplegia (1/3), mild lateral gaze limitation bilaterally (1/3), mild appendicular dysmetria with overshooting (1/3), wide-based gait (1/3), abnormal tandem gait (1/3),dysphagia (1/3),diploplia (1/3), bilateral ptosis with worsening of extraocular movements (1/3), diminished reflexes in the upper and lower limbs (1/3), bilateral papilledema (1/3)	None (2/3), congenital heart disease (1/3)
**Appavu**	12 ± 5.6568	1 (50%)	Right hemiplegia (2/2), language impairment (1/2), aphasia (1/2), lethargy (1/2), limping (1/2), left-sided weakness (1/2), persistent dysarthria (1/2), right facial palsy (1/2), right upper extremity weakness (1/2)	None
**Asif**	18	1 (100%)	Headache, mild photophobia	None
**Balagurunathan**	NR	NR	Encephalopathy with features of cerebellar involvement (1/3), seizures (1/3), headache (1/3), bilateral papilledema (1/3)	NR
**Bauer**	16.5 ± 0.7071	2 (100%)	Altered mental status (2/2), Loss of taste and smell (2/2)	Obesity (BMI, 38) (1/2), high-functioning autism spectrum disorder and anxiety (1/2)
**Becker**	11.25 ± 3.5939	1 (25%)	Headache (2/4), blurry vision (1/4), cranial nerve VI palsy (1/4), bilateral papilledema (1/4), irritability (1/4), nuchal rigidity (3/4), encephalopathy (2/4)	Oppositional defiant disorder (1/4), none (3/4)
**Bektaş**	11	0	Personality changes	None
**Bhatta**	11	1 (100%)	Tonic-clonic seizure	None
**Bhavsar**	16	1 (100%)	Somnolence, headache, confusion, and incoherent speech, seizures, mild to moderate generalized weakness, lethargic, oriented	None
**Biglari**	11	0	Lower limbs paresis, weakness, severe flaccid paraplegia in proximal of lower limbs associated with a positive Lhermitte's sign (electric shock-like sensation down the back triggered by forward bending of head), sensory level at T5, absent deep tendon reflexes and abdominal reflex	None
**Biko**	NR	NR	Altered mental status (1/8)	None(2/8), stroke (1/8), NR (5/8)
**Brum**	0.0472	1 (100%)	Generalized tonic seizure, lethargy	None
**Canham**	18	1 (100%)	Generalized tonic - clonic seizures	Nil known (refugee)
**Caro-Domínguez**	6.972 ± 7.2461 (for children with abnormal findings only	1 (33.33%)	Headache, seizure, persistent drowsiness, decreased level of consciousness, papilledema, confusion, encephalopathy, post cardio-respiratory arrest (all had neurologic symptoms, not specified)	NR (11/12), congenital heart disease (1/12)
**Cecchini**	17	0	Anosmia, occasional parosmia, dysgeusia, no olfactory sensation	NR
**Chen**	8	0	New-onset seizures	NR
**Chiotos**	5	0	Irritability and nuchal rigidity	None
**Coronado Munoz**	6.4771 ± 6.1769	8 (72.7%)	Seizures (3/11), Lethargy (6/11), Headache(3/11), Ataxia (1/11), Left hemiparesis (1/11), LOC (1/11), Hypertonic (1/11), Hypotonic (1/11), Lower extremities weakness (1/11), Right spastic hemiparesis (1/11), Status epilepticus (1/11)	Pulmonary tuberculosis and developed tuberculosis meningitis (1/11), Leukemia (1/11), none (9/11)
**Curtis**	8	1 (100%)	Progressive weakness, intermittent left esotropia and dysconjugate gaze, areflexia	None
**de Miranda Henriques-Souza**	12	0	Headache, progressive, bilateral, and symmetrical motor weakness, tingling and numbness in the inferior limbs, abolished reflexes, flaccid tetraplegia, deep areflexia, and abolished abdominal cutaneous and plantar cutaneous reflexes, dysphagia and dysphonia	None
**de Oliveira**	2	0	Acute-onset divergent strabismus and ptosis in the right eye	None
**Dean**	14	0	Generalized weakness, swallowing difficulty, diffuse tenderness to palpitation over the ribs, shoulders, and upper back, extremity weakness, decreased sensation to light touch on the left lower extremity, "shooting" and "stinging" pains	Obesity

			mostly in upper extremities with sensitivity to touch, numbness of right lower and eye fatigability, sense of impending doom, hyporeflexia in upper extremities, diplopia on horizontal gaze bilaterally and ongoing decreased sensation on lower right face.	
**Divya**	11	0	Headache, lethargy, transient obscurations of vision associated with change in posture	None
**Dugue**	0.125	1 (100%)	Episode of sustained upward gaze associated with bilateral leg stiffening and decreased responsiveness	NR
**Elmas**	15.142 ± 2.0354	4 (57.14%)	Headache (4/7), decreased taste (7/7), Decreased smell (5/7)	NR
**Emami**	1.4525 ± 2.0470	1 (50%)	Status epilepticus (1/2), Altered mental status (2/2), Dysarthria (1/2), bilateral upward plantar reflexes (1/2), seizure (1/2)	Allergy to cow milk (1/2), Low birth weight (1/2)
**Farley**	8	1 (100%)	Left-sided focal seizure with rhythmic movement of the left arm and blinking of the left eye	ADHD, motor tics
**Fenlon Iii**	reported for one patient only 14	0	Fluctuating mental status (2/4), vision complaints and cranial nerve VI palsy (1/4), headache (1/4)	NR
**Foster**	4	1 (100%)	Headaches, tonic-clonic seizure, left-sided hemiparesis	Congenital cerebral ventricular malformation
**Fouriki**	10 ± 4.5825	3 (100%)	Headache (3/3), irritability (3/3), Acute encephalopathy (1/3), meningitis-like symptoms [Phono photophobia/petechiae] (2/3)	None (3/3)
**Fragoso**	newborn	1 (100%)	Focal to bilateral clonic seizures, lethargy and hypotonia , brisk tendon reflexes, no primitive reflexes	NR
**Frank**	15	1 (100%)	Frontal headaches, retro-orbital pain, progressive symmetrical limb weakness, absent deep tendon reflexes	None
**García-Howard**	0.25	0	Convulsions, mild hypotonia	None
**Gaughan**	16	0	Insomnia, anorexia, paranoia, ritualistic behaviors, visual and auditory hallucinations, mutism, little to no voluntary motor activity, motor perseveration with repetitive scissoring movements involving her legs and circular movements involving her arms, bilateral limb rigidity with subtle high frequency tremor	Mild learning needs
**Gaur**	10.5 ± 2.1213	2 (100%)	Headache (1/2), lethargy (2/2), altered mental state (1/2),dysarthia (1/2) and ataxia (1/2)	NR
**Giannantonio**	13	1 (100%)	Mild photophobia, headache, spontaneous horizontal- torsional grade III nystagmus, Transient- evoked acoustic otoemissions	None
**Gulko**	13	0	Headache, speech difficulty, and right upper and lower extremity weakness, anosmia	None
**Gupta Dch**	NR	NR	NR	NR
**Hameed**	NR	NR	Altered neurology and confusion	NR
**Hatipoglu**	13 ± 0	2 (66.66%)	Headache (2/3), Anosmia (3/3), ageusia (2/3), weakness (1/3)	None (1/3), NR (2/3)
**Hossain**	3	1 (100%)	Weakness of lower limbs, reflexes & muscle power was diminished in lower and upper limbs, loss of consciousness and flaccid muscles, encephalopathy	GBS 20 months back and cured at that time without any consequences
**Hosseinpour**	2.583	1 (100%)	Episodic hypotonia, irritability, progressive symmetric weakness in the lower limbs, inability to stand, walk even one step and sit, slurred speech, symmetric and bilateral absent deep tendon reflexes (knee and ankle), pain in the lower limb	None
**Hutchison**	14	1 (100%)	Restless, agitation, and confusion, sleepy, disoriented, aggressive, delusions, inattentive, and unable to follow multistep commands, increased speech latency, flat affect, minimal spontaneous activity, poor eye contact, impairment in attention, concentration, and short-term memory, slowing on rapid alternating movements	None
**Insuga**	7	1 (100%)	Convergent strabismus, blurred vision, binocular diplopia, hypogeusia, and hyposmia	None
**Jillella**	12	1 (100%)	Language impairment, right-sided weakness, dysarthria, aphasia, and right hemiparesis	Asthma
**Khair**	17	0	Acute alteration of mental status, headaches, staring and not speaking with minimal responsiveness to verbal and tactile stimulation, confusion, lethargy, impaired remote memory, slow, hesitant, wide-based gait, agitation	Type I diabetes mellitus (DM) and hypercholesterolemia
**Khalifa**	11	1 (100%)	Unsteady gait and inability to walk or climb stairs associated with tingling sensation felt in both the legs and feet;symmetrical weakness affecting lower limb muscle groups with reduced motor power, hypotonia, lost ankle and knee reflexes, tingling sensations, and an impaired sensation regarding pain and light touch of both feet up to the mid-legs with impaired proprioception, upper limbs reflexes were elicited with reinforcement.	None
**Khan**	11 ± 4	1 (33.33%)	Generalized tonic-clonic seizures(2/3), headache (2/3), delirious (1/3), horizontal nystagmus (2/3), dysarthria (1/3), nuchal rigidity (1/3), features of raised intracranial tension (ICT) and shock (1/3), sudden neurological deterioration (1/3), tremulousness of tongue (1/3)	NR (1/3), right temporal epilepsy on multiple antiepileptic drugs since 5 years of age (1/3), none (1/3)

**Khera**	11	0	Acute onset severe flaccid paralysis, hypotonia, reflexes were absent in ankle, knee and other superficial reflexes and plantar was mute	None
**Khoshnood**	3	0	Dysarthria, wide- spaced gait and falling, irritability, diminished expressive language	Down syndrome (DS), unbalanced atrioventricular (AV) canal status post a 1.5 ventricle repair, a Glenn shunt, atrial septal defect (ASD)/ventricular septal defect (VSD) patch, venovenous collateral ligation resulting in four- chamber physiology with hypoplastic right ventricle, pulmonary hypertension and obstructive sleep apnoea
**Khosravi**	10	0	Headache, facial distortion, tonic movements, loss of consciousness, dysarthria and left-sided hemiparesis	None
**Korkmazer**	10	1 (100%)	Seizures	None
**Krueger**	9.04 ± 7.7274	2 (50%)	Paresthesia (2/4), progressive difficulty to walk (1/4), paraparesis (1/4), with hypoesthesia (1/4), weakness in the lower limbs followed by the upper limb's involvement (1/4), absence of deep tendon reflexes (1/4), quadriparesis (1/4), reduction of superficial and deep sensitivity at the four limb (1/4), headache (1/4), horizontal diplopia (1/4), deviation of the eyes and automatic masticatory movements (1/4)	Hypertensive arachnoid cyst (1/4), NR (3/4)
**Kushwaha**	15	1 (100%)	Headache	NR
**Lazarte-Rantes**	0.75	1 (100%)	Irritability, clonic focal seizure, hyperreflexia, stupor, febrile focal status epilepticus, quadriparesis, hypotonia	None
**Lindan**	8.9036 ± 5.2103	21 (55.26%)	Seizures (7/38), encephalopathy (12/38), dystonic posturing (1/38), headache (10/38), photophobia (1/38), phonophobia (1/38), lower limb weakness (2/38), Upper extremity weakness (1/38), confusion (2/38), facial paralysis (2/38), Dysarthria (1/38), ophthalmoplegia (2/38), gait disturbance (1/38), back pain (1/38), reduced lower limb reflexes (1/38), right hemiparesis (1/38), meningismus (3/38), Lower > upper limb spasticity and brisk DTR's (1/38), reduced weight bearing (1/38), Irritability (1/38), gait impairment (3/38), hyperreflexia (1/38), Gait difficulty (1/38), facial palsy (2/38), myalgias (2/38), blurred vision (1/38), crural paraesthesia & hypoesthesia progressing to T8 sensory level within hours (1/38), neck pain (1/38), cerebellar signs (2/38), weakness (1/38), stupor (1/38), pyramidal signs (1/38), visual hallucinations (1/38), shock (1/38), altered mental status (1/38), neck swelling, weakness (1/38), incoherent speech (1/38), bladder dysfunction (1/38), cerebellar ataxia (1/38), Vertigo (1/38), decreased hearing (1/38), anosmia (1/38), Flaccid paralysis upper and lower limbs (1/38), brainstem dysfunction (1/38), ataxia (1/38), right ptosis (1/38), hypotonia (1/38), four limb dysfunction (3/38)	Mone (32/38), Sickle cell post bone marrow transplant (1/38), Pregnant (1/38), Pes Cavus (1/38), Asthma (2/38), Obese (1/38)
**McLendon**	1.416	0	Worsening weakness, unsteady gait, irritability, subtle right-sided nasolabial fold flattening, significant neck stiffness with a positive Brudzinski's sign, left upper extremity rigidity, right upper extremity paresis, bilateral lower extremity hyperreflexia, and truncal ataxia, autonomic instability, lethargy	None
**Mehra**	13	0	Irritability, agitation, no motor response to painful stimuli and no spontaneous eye-opening, repeated generalized convulsions	NR
**Miller**	6	1 (100%)	None	None
**Ngo**	7.95 ± 7.5742	2(50%)	Febrile status epilepticus (1/4), altered mental status (2/4), headache (1/4), Disorganization (1/4), hyper-sexuality (1/4), hyper-religiosity (1/4), pressured speech (1/4), confusion (2/4), visual hallucinations (2/4), anxiety (1/4), unsteady gait (1/4), nonsensical speech (1/4), auditory hallucinations (1/4), neurogenic bladder (1/4), seizure like episode (1/4), lethargy (1/4), episode of whole-body stiffening (1/4)	Hypotonia and global developmental delay (1/4), prematurity (born at 28 weeks), sickle cell anemia, and gross motor and speech delays (1/4), none (2/4)
**Olivotto**	4.5 ± 1.9148	1 (25%)	Headache (2/4), drowsiness (4/4), irritability (4/4), mood deflection (4/4), sleep disorder (1/4), photophobia (2/4), diffuse limb pain (2/4), oculomotor apraxia (1/4), lower limb weakness and areflexia (1/4), gait disorder (1/4), speech disorder (2/4), Generalized tonic-clonic seizures (1/4), hyporeactivity (1/4), meningism (1/4)	NR
**Orman**	8.835 ± 6.9073	12 (60%)	Impaired consciousness (7/20), seizures (4/20), status epilepticus (2/20), headache (3/20), focal neurologic findings on examination (2/20), meningeal signs on examination (1/20), transient episode of aphasia (1/20)	Epilepsy (1/20), Sickle-celldisease (2/20), Obesity (2/20), Hemophilia-C (1/20), Overweight (1/20), SturgeWeberSyndrome (1/20), Autism (1/20), none (11/20)
**Oualha**	16	1 (100%)	Aseptic meningitis associated with stupor, right hemiparesis	None
**Palabiyik**	NR	NR	Epilepsy (14/21), neck stiffness (6/21), and inability to walk (1/21), headache, hallucination (not specified)	None (21/21)

**Paterson**	16.5 ± 0.7071	0	Diplopia (1/2), headache with fea tures of raised ICP (1/2), Evolving cranial nerve signs (1/2), progressive bilateral failure of abduction (1/2), weakness of left orbicularis oculi (1/2), Seizures (1/2), Low conscious level (1/2), possible cortical visual impairment (1/2), increased tone and clonus (1/2)	Menorrhagia (1/2), Cornelia de Lange syndrome; epilepsy; dysmelia; hypertension; visual disturbance, GORD; Nissen fundoplica tion; gastrostomy (1/2)
**Penner**	NR	NR	Headaches, dysarthria or dysphonia, visual or auditory hallucin ations , unsteady gait, and seizures, encephalopathy, delirium, ataxia, peripheral neuropathy, abnormal eye movements, saccades, facial asymmetry, weakness (all had neurologic symptoms, not specified)	NR
**Powers**	11	1 (100%)	New onset gaze preference towards the left, along with opsoclonic eye movements and global aphasia, rubral tremor	History of FARS2-related combined oxidative phosphorylation deficiency type 14, spastic paraparesis, and mild developmental delay
**Ray**	8.8491 ± 4.4272	25 (59.52%)	Ageusia (1/42), encephalopathy (26/42), headache (11/42), movement disorder (1/42), ataxia (9/42), cranial neuropathies (1/42), facial weakness (3/42), R eye ptosis (1/42), meningism (3/42), neck pain (1/42), back pain (2/42), enuresis (1/42), relapse R optic neuritis (1/42), loss of motor skills (1/42), hoarse voice (1/42), areflexia (6/42), weakness (12/42), unable to weight bear (1/42), seizure (6/42), urinary incontinence (1/42), reduced power (1/42), lethargy (2/42), photophobia (1/42), hyperacusis (1/42), excessive sleepiness (1/42), Status Epilepticus (6/42), aggression (1/42), selfharm (1/42), auditory hallucinations (3/42), visual hallucinations (6/42), behavioural changes (5/42), involuntary movements of the R shoulder (1/42), chorea (2/42), dyskinesia & limb posturing (1/42), anosmia (1/42), Unable to walk (1/42), brain stem signs (1/42), L hemiplegia (1/42), myelopathy (1/42), urinary retention (1/42), bilateral foot drop (1/42), R lower limb neuropathic pain (1/42), dysphasia (1/42), dysphonia (2/42), left Horner's syndrome (1/42), R upper limb hypertonia (1/42), subtle dysmetria bilaterally (1/42), cognitive regression (1/42)	post neonatal meningitis ventriculomegaly (1/42), neuroblastoma (1/42), epilepsy (4/42), ASD (4/42), expremature infant (2/42), LD (1/42), previous L basal ganglia stroke (1/42), SSD (2/42), Type 1 Diabetes Mellitus (1/42), Asthma (1/42), none (31/42)
**Riollano-Cruz**	5	1 (100%)	None	None
**Saeed**	3	1 (100%)	Tonic colonic convulsions	None
**Sahu**	5	1 (100%)	Generalized seizures, bilateral lower limb weakness, meningeal irritation (positive Kernig's sign, and neck rigidity), hyporeflexia in both legs, truncal weakness, superficial abdominal reflexes were absent as well and plantar reflexes were bilaterally extensor	NR
**Salman**	NR	0	Drowsiness and meaningless speech	NR
**Sánchez-Morales**	12.166 ± 5.1542	3 (50%)	Headache (2/6), Diplopia (1/6), ocular pain (2/6), and diminished visual acuity (2/6), left VI cranial nerve paresis (1/6), myalgias (1/6), Altered behavior and mental status (2/6), seizures (2/6), insomnia (1/6), orolingual dyskinesias (1/6), aphasia (2/6), left hemiparesis (2/6), Irritability (2/6), weakness (1/6), Ataxia 91/6)	None (4/6), Aortic coarctation (1/6), Acute myeloblastic leukemia M2 (1/6)
**Sandoval**	6.2 ± 5.0199	3 (60%)	Febrile seizure (1/5), seizure (1/5), psychomotor agitation (1/5), insomnia (1/5), visual hallucinations (1/5), headache (3/5), proximal generalized weakness (1/5), hyporeflexia (1/5), orthostatic intolerance (1/5), blurry vision (1/5), papilledema VI right cranial nerve palsy (1/5), asymmetric mild paraparesis (1/5), Bilateral ankle clonus (1/5), left Babinski sign (1/5), ophthalmoparesis (1/5), facial diparesis (1/5), acute progressive ascending flaccid tetraparesis (1/5), areflexia (1/5)	A previous single febrile seizure (1/5), Atopic dermatitis (1/5), TBI with a previous skull fracture (1/5), none (2/5)
**Sarigecili**	7	1 (100%)	Ataxia, widebased gait, Deep tendon reflexes could not be elicited, somnolence, seizures, choreiform movements in the hands and feet, tongue protrusion, bruxism, lip smacking, agitation, catatonia, echolalia	None
**Schiff**	0.333	0	Bulging anterior fontanelle	NR
**Shala**	14	1 (100%)	Right side hemiplegia and aphasia	None
**Shenker**	12	1 (100%)	Trismus, loss of smell and taste, difficulty swallowing, waxing and waning episodes of rapid, tangential speech, hyperactivity, and emotional lability, seizures, episode of altered mental status, agitation	None
**Sofijanova**	0.75	NR	Tonic-clonic seizures, disturbed consciousness, weakened reaction to painful stimuli	NR
**Sofuoğlu**	11	0	Headache, neck pain, neck stiffness, positive Kernig and Brudzinski signs, alteration of consciousness, diplopia, Left abducens paralysis and bilateral grade 3 papilledema	None
**Tiwari**	9	0	Headache, progressive weakness on the right side of body, upper motor neuron type right sided seventh cranial-nerve palsy, complete hemiplegia, brisk deep tendon reflexes, and extensor plantar response on the right	None

**Tomar**	13	1 (100%)	Lethargy, headache, clumsiness in both hands, slurring of speech, swaying side to side while walking, mild nystagmus, dysarthria, impaired finger nose testing, dysdiadochokinesia, impaired tandem walk	None
**Ucan**	NR	NR	Lethargy, confusion, irritability, encephalopathy and seizures, cerebellar ataxia, altered mental status and gait disturbance (all had neurologic symptoms, not specified)	NR
**Urso**	5	0	Altered mental status, increased irritability, sleepiness, lack of energy, and lethargy	None
**Vivanti**	neonate	1 (100%)	Irritability, poor feeding, axial hypertonia, and opisthotonos	NR
**Vraka**	5.54 ± 6.3073	0	Altered consciousness (1/2), seizures(1/2), lethargy (1/2), ageusia (1/2), headache (1/2), malaise (1/2), stopped speaking (1/2), stopped mobilising (1/2), stopped using right arm (1/2), hypertonia (1/2), brisk reflexes (1/2), right-sided Babinski (1/2), sluggish pupils (1/2), autonomic disturbance (1/2)	None (2/2)
**Wang**	17	1 (100%)	Loss of smell and taste.	Appendectomy five years ago
**Yan**	newborn	2(40%)	Lethargy (2/5)	31-week prematurity (1/5), none(4/5)
**Yildiz**	0.133	1 (100%)	Seizure	None
**Zain**	1.916	0	Drooping of right eye and right side of mouth	None
**Zubarioglu**	0.75	0	Altered level of consciousness and acute loss of acquired motor skills, increased tendency to sleep, lethargic, macrocephaly, generalized hypotonia, complete loss of voluntary movements, Mild choreoathetoid movements, especially marked on extremities	Glutamic aciduria type 1

NR = not reported; SD = standard deviation

Supplementary Table.3 Imaging Details

First Author's Name	Imaging modality (n)	MR Tesla	MR protocol	Number of neuroradiologist reviewers and experience
**Abel**	CT, MRI	NR	ADC, DWI, T2-FLAIR	NR
**Akçay**	MRI (2), MRA (1)	NR	CE-FLAIR, DWI,ADC	NR
**Akhondian**	MRI (5), CT (1)	NR	T2WI, FLAIR, DWI, ADC	NR
**Aksu Uzunhan**	MRI (2)	1.5 T	DWI, T2WI	1 radiologist (NR)
**Al Haboob**	CT, MRI, MRV, MRA	NR	FLAIR, T2WI	NR
**Aljomah**	CT (2), angiogram (1), MRI (2)	NR	NR	NR
**Appavu**	MRI (2),MR VWI(2),MRA (2),CT (1)	NR	CE-MR VWI	NR
**Asif**	CT, venogram	/	/	NR
**Balagurunathan**	NR	NR	NR	NR
**Bauer**	MRI (2)	NR	FLAIR	NR
**Becker**	CT (4), MRI (2), MRV (1), MRA (1)	NR	NR	NR
**Bektaş**	MRI	NR	T2WI, DWI, ADC	NR
**Bhatta**	CT	/	/	NR
**Bhavsar**	CT	/	/	NR
**Biglari**	MRI	NR	CE-T2WI , CE- T1WI	NR
**Biko**	MRI (7), CT (6)	NR	NR	2 radiologists (10 and 3 years of experience)
**Brum**	MRI	NR	T1WI, T2WI, DWI, ADC	NR
**Canham**	CT, venography	/	/	NR
**Caro-Domínguez**	MRI (9), CT (3)	NR	FLAIR, DWI, ADC	2 radiologist (6 and 7 years of experience)
**Cecchini**	MRI	3 T	T2WI, FLAIR	NR
**Chen**	MRI	NR	T2WI, DWI	NR
**Chiotos**	CT	/	/	NR
**Coronado Munoz**	MRI (1), CT (10)	NR	NR	NR
**Curtis**	CT, MRI	NR	T1WI, CE-T1WI	NR
**de Miranda Henriques-Souza**	MRI	NR	ADC, DWI, FLAIR, CE-T1WI, SWI, T2WI	NR
**de Oliveira**	NR	NR	NR	NR
**Dean**	MRI	NR	CE-T1WI, T2WI	NR
**Divya**	MRI, MRV, OCT, US	NR	NR	NR
**Dugue**	MRI	NR	NR	NR
**Elmas**	MRI (7)	NR	DWI	1 radiologist (NR)
**Emami**	CT (1), MRI (1), US (1)	NR	NR	NR
**Farley**	CT	/	/	NR
**Fenlon Iii**	MRI (4)	NR	FLAIR, DWI	1 radiologist (fellow)
**Foster**	CT, MRI, MRV, MRA	NR	DWI, FIESTA	NR
**Fouriki**	MRI (3)	NR	NR	NR
**Fragoso**	MRI	NR	T1WI, T2WI, DWI, ADC, SWI	NR
**Frank**	MRI	NR	NR	NR
**García-Howard**	CT, MRI	1.5 T	NR	NR

**Gaughan**	MRI	NR	T1WI, T2WI, FLAIR, DWI, CE-T1WI	NR
**Gaur**	MRI (2)	NR	T2WI, DWI, ADC	NR
**Giannantonio**	MRI	NR	NR	NR
**Gulko**	CT, MRI, MRA, ,MR VWI, US	3 T	DWI, ADC, FLAIR, TOF MRA, CE 3D T1 isotropic turbo spin-echo acquisition and black-blood sequences	NR
**Gupta Dch**	CT (8), USG (1)	/	/	NR
**Hameed**	CT (4), MRI (3)	NR	NR	2 radiologists (5 and 10 years of experience)
**Hatipoglu**	MRI (3)	1.5 T	CISS-GE, FLAIR and CE- fat-suppressed T1WI	1 neuroradiologist (NR)
**Hossain**	MRI	NR	NR	NR
**Hosseinpour**	CT, MRI	NR	T1WI, T2WI, FLAIR	NR
**Hutchison**	MRI	NR	NR	NR
**Insuga**	OCT, MRV	NR	NR	NR
**Jillella**	CT, CTA, MRI	NR	DWI, ADC	NR
**Khair**	CT, MRI, MRV	NR	DWI	NR
**Khalifa**	MRI	NR	CE-T1FS	NR
**Khan**	MRI (2), CT (1)	NR	CEMRI, T2WI, FLAIR, DWI, T1FS	NR
**Khera**	MRI	NR	CEMRI, T2WI, FLAIR, DWI, T1FS	NR
**Khoshnood**	MRI	NR	GRE, DWI, FLAIR	NR
**Khosravi**	MRI, MRA, MRV	NR	NR	NR
**Korkmazer**	MRI	NR	T2WI, FLAIR, DWI, ADC	NR
**Krueger**	MRI (4), MRA (1), angiograph (1)	NR	CE-T1WI, T2WI	NR
**Kushwaha**	MRI	NR	NR	NR
**Lazarte-Rantes**	MRI	NR	T2WI, T1IR, DWI, ADC, SWI	NR
**Lindan**	MRI (38), MRA (1), CT (2)	NR	T2WI, FLAIR, DWI, CE-T1WI, ADC, SWI, FSE- T2	5 neuroradiologists (100 years of combined experience)
**McLendon**	MRI	NR	T2WI, FLAIR	NR
**Mehra**	MRI	NR	T2WI, FLAIR, DWI	NR
**Miller**	MRI	NR	CE - T2WI	NR
**Ngo**	MRI (4), MRA (2), CT (1)	NR	T2WI, DWI	NR
**Olivotto**	MRI (4)	NR	T1WI, T2WI, FLAIR, DWI, ADC	NR
**Orman**	CT (17), MRI (17),MRV (2), and MRA (7)	NR	FLAIR	2 neuroradiologists (9 and 10 years of experience)
**Oualha**	MRI	NR	NR	NR
**Palabiyik**	MRI (21)	NR	DWI	1 radiologist (11 years of experience)
**Paterson**	MRI (2)	NR	NR	NR
**Penner**	MRI (16)	NR	NR	NR
**Powers**	MRI	NR	DWI	NR
**Ray**	MRI (38), CT (10), MRA (1)	NR	T2WI, FLAIR, T1WI, DWI, ADC	1 neuroradiologist (NR)
**Riollano-Cruz**	CT	/	/	NR
**Saeed**	CT, MRI	NR	NR	NR
**Sahu**	MRI	NR	FLAIR, T2WI	NR
**Salman**	MRI	NR	NR	NR
**Sánchez-Morales**	MRI (5), CT(1)	NR	NR	NR
**Sandoval**	CT (4), MRI (2)	NR	FLAIR, CE_T1WI, T2-weighted STIR, T2-weighted SE	NR
**Sarigecili**	MRI	NR	NR	NR
**Schiff**	CT	/	/	NR
**Shala**	MRI,MRA, CTA, US	NR	ADC, DWI, FLAIR	NR
**Shenker**	CT, MRI, MRA, MRV	NR	NR	NR
**Sofijanova**	CT	/	/	NR
**Sofuoğlu**	MRI, MRV	NR	T2WI, FLAIR, ADC	NR
**Tiwari**	CT, CTA	/	/	NR
**Tomar**	MRI	NR	FLAIR	NR
**Ucan**	MRI (3), CT (1)	NR	FLAIR, T2WI, DWI, ADC	2 radiologists (5–15 years of experience)
**Urso**	MRI	1.5 T	FSE, DWI, T1WI, T2WI,FLAIR, CE-T1WI	NR
**Vivanti**	MRI, US	NR	T1WI, DWI	NR
**Vraka**	CT (2), MRI (2)	NR	T2WI, DWI	NR
**Wang**	MRI	NR	NR	NR
**Yan**	MRI (5)	1.5 T	T1WI, T2WI, DWI	1 radiologist (NR)
**Yildiz**	US, MRI	1.5 T	DWI, ADC, T1WI, T2WI	NR
**Zain**	MRI	NR	CE-T1WI	NR
**Zubarioglu**	MRI	NR	T2WI	NR

NR = not reported; DWI = diffusion-weighted imaging; ADC = Apparent diffusion coefficient; T1WI = T1-weighted imaging; T2WI = T2-weighted imaging; CE = contrast-enhanced; FLAIR = fluid attenuated inversion recovery; SWI = susceptibility-weighted imaging; STIR = Short Tau Inversion Recovery; FSE = Fast Spin Echo; T1FS = T1-Weighted Fat-Suppressed; VWI = vessel wall imaging; TOF = Time-Of-Flight.

Supplementary Table 4. Detailed Neuroimaging Findings in children with COVID-19 (Case-report and case-series)

First Author's Name	Neuroimaging findings
**Abel**	Restricted diffusion in the bilateral lateral thalamic nuclei
**Akçay**	Pathologic signal changes in cortical and subcortical areas in bilateral frontoparietal regions, lesions showed contrast enhancement after contrast enhancement, and marked diffusion restriction (1/2); pathologic signal increases in the bilateral temporal region in the hippocampal areas, bilateral thalamus, putamen and deep white matter (1/2)
**Akhondian**	Hyperintensities in the right frontal and parietal regions with restriction diffusion in the affected area which reveals acute stroke in the bifrontal and biparietal lobes (1/5); hyperintense signals in bilateral dentate nuclei, pons, bilateral thalamic regions, and midbrain (1/5); restriction diffusion in the left centrum semiovale region (1/5); Several Cortical and Subcortical Involvement (1/5); right sentrum semioval is affected, restriction diffusion in the right posterior periventricular region and thalamus, intraventricular hemorrhage (1/5)
**Al Haboob**	Nonspecific high signal intensity surrounding the occipital horns, Bilateral cortical and subcortical patchy high signal intensity affecting parieto occipital areas with mild involvement of frontal areas (at watershed zones) in both thalami, hypoplastic left lateral sinus, other venous sinuses, and deep venous system are patent
**Aljomah**	Thickening and enhancement of the nerve roots of the conus medullaris, cauda equina, exiting nerve roots exiting from L5 to S2 (1/3); Rotated vermis, hypoplastic corpus callosum , and left intraventricular hemorrhage, with a small left frontal hemorrhage (1/3)
**Appavu**	Small completed infarctions in the middle cerebral artery (MCA) territories bilaterally, complete occlusion of proximal M1 segment of the left MCA, thick concentric mural enhancement of supraclinoid LICA (1/2); complete left MCA territory infarction, irregularity of left M1 (suggestive of arteritis), and occlusion of left MCA bifurcation, worsening edema and increased midline shift (1/2)
**Asif**	Hyperdense internal cerebral veins, filling defects throughout the sigmoid and transverse sinuses bilaterally, extending into straight and superior sagittal sinuses
**Bauer**	Unchanged subcortical linear FLAIR hyperintensity in mid-left temporal lobe attributed to gliosis surrounding a developmental venous anomaly (not related to COVID-19)
**Becker**	Restricted diffusion of optic nerve sheaths, flattening of the posterior sclera, and eversion of the optic discs, consistent with papilledema, flattening of the left transverse and sigmoid sinuses (1/4); cerebral edema (1/4);
**Bektaş**	Hyperintensity and restricted diffusion in the splenium of the corpus callosum
**Bhatta**	Normal
**Bhavsar**	Normal
**Biglari**	High signal intensity at T3-T9 segments of the spinal cord associated with spinal cord swelling at T3-T6 segments, spinal cord swelling and a central high signal area observed in the both levels of the spinal cord, mild heterogeneous patchy enhancement in the involved areas
**Brum**	Two small foci of restriction in the left frontal subcortical white matter with restricted diffusion compatible with acute ischemic lesions
**Canham**	Normal
**Cecchini**	Normal
**Chen**	Extensive T2 hyperintensity centered at bilateral basal ganglia, extending to the frontal white matter, external and internal capsules, corpus callosum, thalami, insula, as well as the cerebellar hemispheres, brainstem and the spinal cord; persistent diffusion restriction in the affected white matter throughout much of the central white matter tracts, cerebral peduncles, corticospinal tracts and cerebellar white matter; vacuolating necrosis in bilateral basal ganglia.
**Chiotos**	Diffuse cerebral edema
**Curtis**	Abnormal enhancement of the posterior nerve roots from the T11 level through the cauda equina
**de Miranda Henriques-Souza**	Extensive bilateral and symmetric restricted diffusion involving the subcortical and deep white matter, focal hyperintense lesion in the splenium of the corpus callosum with restricted diffusion, longitudinally extensive cervical myelopathy involving both white and gray matter
**de Oliveira**	Normal
**Dean**	Asymmetric increased enhancing abnormality in bilateral oculomotor nerves, left greater than right, Almost symmetric enhancing abnormality of bilateral trigeminal nerves with associated mild thickening, Subtle patchy enhancement abnormality in the facial and vestibulocochlear nerve complexes in bilateral internal auditory canals, Patchy enhancing abnormalities in bilateral vagus nerves, right greater than left; smooth enhancing abnormality in cauda equina (ventral greater than dorsal) with subtle thickening of nerve root; there was mild swelling of the right hippocampal head with abnormal T2 signal.
**Divya**	Increased thickness of peripapillary retinal nerve fiber layer (RNFL) in both eyes; increased optic nerve sheath diameter
**Dugue**	Normal
**Farley**	Normal
**Foster**	New diffusion restriction indicative of cytotoxic edema secondary to infarction throughout both hemispheres, thalami, and basal ganglia; Variable degrees of focal narrowing and irregularity are noted along the courses of the distal left carotid artery, left M1 and M2 and right postbifurcation M1 segments of the middle cerebral arteries, right A1 segment of anterior cerebral artery, and left P2 and right P1 segments of the posterior cerebral arteries
**Fouriki**	Localized meningeal enhancement sulcus centralis and postcentral region (1/3)
**Fragoso**	Tenuous signal change areas in the bilateral white matter of the cerebral hemispheres, with sparse foci of restricted diffusion, without associated hemorrhage; marked diffusion restriction throughout the corpus callosum and, less prominently, along the corticospinal tracts, from the posterior arms of the internal capsules to the bulb pyramids
**Frank**	Normal
**García-Howard**	Normal

**Gaughan**	Two tiny, punctate T2/FLAIR hyperintensities in the centrum semiovale bilaterally, with no diffusion restriction or contrast enhancement
**Gaur**	Small, ovalshaped, T2-weighted hyperintense lesion in the splenium of the corpus callosum that exhibited restricted diffusion (1/2); similar but more extensive abnormal T2-weighted hyperintense signal and restricted diffusion in the entire corpus callosum and frontoparietal cerebral white matter. This had a dorsal predilection, with greatest in-volvement of the splenium, peri-atrial white matter, and posterior frontal and parietal centrum semiovale (1/2)
**Giannantonio**	Normal
**Gulko**	Left frontal hypodensity concerning for ischemic infarct; foci of restricted diffusion and cytotoxic edema within the left middle cerebral artery vascular territory, consistent with acute infarcts; focal segment of moderate stenosis within the left M1 middle cerebral artery; wall thickening and marked, concentric contrast enhancement at the site of stenosis
**Hameed**	Large anterior and middle cerebral artery infarct in a child, likely secondary to extracorporeal membrane oxygenation
**Hatipoglu**	Normal
**Hossain**	Normal
**Hosseinpour**	Normal
**Hutchison**	Normal
**Insuga**	Increased retinal nerve fiber layer thickness
**Jillella**	Alberta Stroke Program Early CT Score of a 9, left internal carotid artery occlusion in the supra-clinoid segment, restricted diffusion correlating with a small acute border-zone infarction in the left middle cerebral artery territory, hypointense area correlating with a small acute border-zone infarction in the left middle cerebral artery territory.
**Khair**	Normal
**Khalifa**	Cauda equina nerve root enhancement
**Khan**	Diffuse cerebellar swelling with T2/FLAIR hyper intensity .These areas showed diffusion restriction on DW images and post-contrast enhancement with mass effect suggestive of acute cerebellitis (1/3); Area of gyral swelling with hyper intensity in left frontal lobe on T2WI. Multiple smaller areas of hyper intensity are seen in the bilateral frontal and parietal lobes. On post-contrast T1FS image, these show enhancements. Findings favored encephalomyelitis (1/3); small hypo-dense area involving subcortical white matter in the right frontal lobe suggestive of infarct and asmall intracerebral hematoma in left frontal lobe with mild perilesional edema. (1/3)
**Khera**	Abnormal hyperintense signal of the lower dorsal cord extending from D7 to D10 vertebra levels without any cord expansion, enhancement of the cauda equina nerve roots without enhancement of the lower dorsal cord, focal subcortical hyperintense lesions with restricted diffusion at right parietal white matter without any perilesional edema
**Khoshnood**	Left cerebellar hemisphere haemorrhagic conversion, restricted diffusion in the left cerebellum consistent with cerebrovascular accident (CVA), signal prolongation in multiple anterior vascular distributions, restricted diffusion in distal cortical areas consistent with embolic stroke, signal prolongation along cerebellar infarction, signal prolongation along the periventricular zones, there is limited oedema surrounding the areas of thromboembolic infarct, aside from the left cerebellar hemisphere, likely due to evidence of haemorrhagic conversion and subsequent vasogenic oedema
**Khosravi**	Acute infarction in the right putamen, globus pallidus, and the posterior part of the insula, small focal narrowing within the right middle cerebral artery (MCA)
**Korkmazer**	Multiple cortico-subcortical increased signal intensity in the bilateral posterior hemispheric regions, especially in right occipital, temporal and parietal lobes, vasogenic edema consistent with PRES
**Krueger**	Bilateral contrast enhancement in the cranial nerves VII/VIII complex, inside the internal auditory canal, contrast enhancement in the anterior roots of the medullary cone (1/4); thickening and contrast enhancement on the anterior surface of the medullary cone (1/4)
**Lazarte-Rantes**	Necrosis of both the thalamus and at the cortico-subcortical junction of both parietal and occipital lobes with a lack of contrast enhancement, restriction in the posterior limb of the internal capsule, cortico-subcortical junction of the occipital lobes, the posterior body of the corpus callosum, and corona radiate, A little dot of hemorrhage was visualized in the frontal lobe at the initial exam as a blooming artifact
**Lindan**	Multifocal T2 hyperintensity throughout bilateral thalami (1/38); Confluent areas of T2 hyperintensity and restricted diffusion in the central gray, pons, and subcortical white matter, and Splenial lesion (1/38); Diffuse leptomeningeal enhancement. Patchy T2 hyperintensity of cerebral white matter and cerebellum; Cortical, thalamic and splenial signal abnormalities (1/38); Extensive patchy white matter and basal ganglia T2 hyperintensities, Associated mass effect and mild enhancement R frontal lobe, Long segment mildly expansive central cord T2 hyperintensity (1/38); Expansile T2 hyperintense signal from obex to mid-thoracic cord with mild enhancement (1/38); Enhancement of CN VII and XII (1/38); Enhancement of CN V-VIII, Enhancement of cauda equina and cervical nerve roots (1/38); Small left frontal infarction, minimal leptomeningeal enhancement. Diffuse cerebral edema and absent cerebral arterial flow on MRA; Central gray abnormal signal and extensive cervical cord edema (1/38); Scattered microhemorrhages and small infarcts in the superficial and deep white matter and in the CC (1/38); Enhancing choroid plexus in left lateral ventricle & bilateral foramina of Luschka. Ependymal enhancement lateral ventricle with DWI restricting material in occipital horn. Small enhancing cerebral abscesses with reduced diffusion (1/38); Sinusitis, rapidly progressive cavernous sinus thrombosis, ophthalmic vein thrombosis, leptomeningitis, CN enhancement, multivessel vasculitis with vessel wall enhancement. Multiterritory ischemic infarction (1/38); Subcortical edema bilateral occipital, posterior temporal, frontal lobes (1/38); T2 hyperintensity brainstem, Long segment cord T2 hyperintensity with central gray matter predominance (1/38); Punctate and linear T2 hyperintense foci and enhancement in subcortical white matter, Long segment T2 hyperintensity with central gray matter predominance and patchy enhancement. Enhancement of cauda equina (1/38); Long segment T2 hyperintensity from the obex through the mid thoracic cord, with central predominance (1/38); Patchy T2 hyperintensity white matter and basal ganglia, brainstem, cerebellar peduncles (1/38); Enhancement and thickening of cauda equina & cervical spinal nerve roots (1/38); Enhancement CN III, V-VII, XII, Enhancement and thickening of cauda equina. Enhancement of spinal nerve roots (1/38); Enhancement of CN III, VI-VIII, Enhancement of cauda equina (1/38); Complete thrombosis superior sagittal sinus. Bilateral haemorrhagic venous infarctions left frontal and right parietal lobes (1/38); T2 hyperintensity cerebral white WM.<br>Focal CC and splenial lesions CC T2 hyperintensity andrestricted diffusion (1/38); T2 hyperintensity cerebral white WM. Myositis: Enhancement of upper cervical musculature (1/38); T2 hyperintensity cerebral WM. Splenial lesion T2 hyperintensity and restricted diffusion Myositis: Upper cervical soft tissues & muscles of mastication (1/38); T2 hyperintensity hypothalamus. Neuritis: Bilateral CN VII (1/38); T2 hyperintensity hypothalamus. Focal T2 hyperintensity in T-cord with central predominance (1/38); T2 hyperintensity cerebral WM. Splenial lesion T2 hyperintensity and restricted diffusion (1/38); T2 hyperintensity cerebral WM. Splenial lesion T2 hyperintensity and restricted diffusion Innumerable microthrombi cerebrum, brainstem, cerebellum (1/38); Splenial lesion T2 hyperintensity and restricted diffusion Focus of restricted diffusion right ventricle occipital horn. Punctate enhancement left temporal WM. Myositis: Suboccipital soft tissues (1/38); Splenial lesion with T2 hyperintensity. Enhancement left CN III (1/38); Splenial lesion T2 hyperintensity and restricted diffusion Myositis: Suboccipital soft tissues (1/38); Enhancement and thickening of the cauda equina (1/38); Left midbrain infarction. Thrombus anterior perforator artery with arterial wall enhancement (1/38); Loss of normal labyrinthine T2 signal and abnormal enhancement (1/38); Enhancement & thickening cauda equina (1/38); Enhancement CN III, V, VII; Enhancement cauda equina (1/38); Swollen cerebellar hemisphere without restricted diffusion. Enhancement of bilateral CN VII (1/38); Patchy T2 hyperintensities in cerebral WM, thalami, brainstem and cerebellum.

	Associated foci of enhancement and restricted diffusion. Enhancement CN III, Long segment cord T2 hyperintensity with central gray predominance and without enhancement (1/38); T2 hyperintense lesions cerebral cortex and thalamus. (1/38).
**McLendon**	Multifocal hyperintense T2 fluid-attenuated inversion recovery signals in bilateral subcortical and periventricular white matter without contrast enhancement
**Mehra**	Extensive lesions with altered T2 and FLAIR signals at gray and white matter junction of both cerebral hemispheres with mild associated enhancement, diffuse cortical swelling with diffusion restriction
**Miller**	Mild kinking of the intraorbital optic nerves and mildly distended optic nerve sheaths bilaterally
**Ngo**	Few small punctate T2-weighted nonspecific hyperintense lesions (1/4); nonspecific restricted diffusion in the splenium of the corpus callosum (1/4)
**Powers**	Restricted diffusion in the periaqueductal grey matter and dorsal midbrain
**Saeed**	Severe brain edema, intracerebral hemorrhage in the right occipital lobe
**Sahu**	Multifocal T2/FLAIR hyperintensities in right frontal lobe, bilateral periventricular regions, and left sub cortical white matter, longitudinal, patchy T2 hyperintense lesions in dorsal column extending from D1 to D12 vertebra level along with cord expansion
**Sánchez-Morales**	Optic nerve hyperintensities (2/6); AIS in left frontal lobe (1/6); AIS in watershed areas (1/6)
**Sandoval**	Subtle nodular hypodensity is present in the right frontal subcortical white matter (1/5); Multiple demyelinated plaques with perivenular distribution are present, Subtle nodular enhancement is evident with gadolinium, Demyelinated plaques are present in the left temporal lobe and in the left cerebral peduncle with intense enhancement, Demyelinated plaques in both cerebellar peduncles, with contrast enhancement, Small demyelinated plaque can be seen in the anterior medulla with lineal enhancement (1/5)
**Sarigecili**	Normal
**Schiff**	Normal
**Shala**	Acute brain ischemia in the territory of the medial cerebral artery, a slight increase of the pulsatility index (PI= 1.18) was observed, suggesting distal resistance due to occlusion ofthe terminal arterial branches ofthe left medial cerebral artery (MCA), a hyperintense signal and edema of the caudate nucleus head, putamen, and parts of the external capsule and insula on the left side
**Shenker**	Normal
**Sofijanova**	Enlargement of the lateral ventricles, with intraventricular masses and pronounced internal hydrocephalus
**Sofuoğlu**	Hyperintensity and restricted diffusion in the splenium of the corpus callosum, optic nerve head protrusion and globe flattening
**Tiwari**	Hypodensities in corpus callosum, left caudate, putamen and bilateral thalami , with mild compression over the left lateral ventricle; ubstantial stenosis of the anterior cerebral artery, stenosis of bilateral supraclinoid internal carotid artery segments, diffuse stenosis of M1 segment of the right MCA and diffuse narrowing of the M2 and M3 segments of both MCA; multifocal narrowing involving both internal carotid arteries, anterior cerebral artery, and right MCA, diffuse narrowing of the segment M2 and M3 of both MCAs ; on the left side there is sudden tapering of the MCA at the M1–M2 junction
**Tomar**	Normal
**Urso**	Presence of a hyperintense focal lesion in the splenium of the corpus callosum, which appeared slightly swollen, and an additional area with the same signal characteristics in the right parietal subcortical area; lesions appeared hyperintense, but did not show any contrast enhancement
**Vivanti**	Hyperintensities of the periventricular and subcortical frontal or parietal white matter
**Vraka**	Bihemispheric white matter hypodensities, posteriorly more than anteriorly, bilateral T2 hyperintensities of the subcortical white matter of all the brain and splenium ofthe corpus callosum with associated diffusion restriction and signal change in thalami and pons (1/2); bilateral T2 hyperintensity of the basal ganglia and parasagittal frontal lobes, anterior limb of the left internal capsule, insula, and subcortical white matter regions (1/2)
**Wang**	Normal
**Yildiz**	Periventricular echogenicity in the periventricular and deep white matter, restricted diffusion in the periventricular white matter, subcortical white matter, corpus callosum, internal capsule, optic radiation and posterior thalami
**Zain**	Abnormal enhancement along the canalicular component of the right seventh cranial nerve extending to the first genu, bilateral enhancement along the tympanic segment
**Zubarioglu**	Opercular frontotemporal atrophy, bilateral subcortical and periventricular white matter, bilateral basal ganglia involvement, and thalamic hyperintensities

Supplementary Table 5. Summary of Neuroimaging Findings in Pediatric COVID-19 Studies

Author	Year	Neurovascular findings	ADEM-like lesions	Encephalitic pattern	Myelitis	Transient splenial lesions	Others	Total
**Caro-Dominguez**	2021	0	0	0	0	1	1	12
**Orman**	2021	1	0	0	0	0	1	20
**Palabiyik**	2021	0	1	1	1	6	1	21
**Penner**	2021	3	0	0	0	4	5	16
**Ray**	2021	3	4	10	2	4	7	42

PRISMA 2020 checklist

Section and Topic	Item #	Checklist item	Location where item is reported
**TITLE**			
Title	1	Identify the report as a systematic review.	Page 1
**ABSTRACT**			
Abstract	2	See the PRISMA 2020 for Abstracts checklist.	Page 1
**INTRODUCTION**			
Rationale	3	Describe the rationale for the review in the context of existing knowledge.	Page 2-3-4
Objectives	4	Provide an explicit statement of the objective(s) or question(s) the review addresses.	Page 4
**METHODS**			
Eligibility criteria	5	Specify the inclusion and exclusion criteria for the review and how studies were grouped for the syntheses.	Page 6-7
Information sources	6	Specify all databases, registers, websites, organisations, reference lists and other sources searched or consulted to identify studies. Specify the date when each source was last searched or consulted.	Page 5-6
Search strategy	7	Present the full search strategies for all databases, registers and websites, including any filters and limits used.	Page 5-6
Selection process	8	Specify the methods used to decide whether a study met the inclusion criteria of the review, including how many reviewers screened each record and each report retrieved, whether they worked independently, and if applicable, details of automation tools used in the process.	Page 6
Data collection process	9	Specify the methods used to collect data from reports, including how many reviewers collected data from each report, whether they worked independently, any processes for obtaining or confirming data from study investigators, and if applicable, details of automation tools used in the process.	Page 7
Data items	10a	List and define all outcomes for which data were sought. Specify whether all results that were compatible with each outcome domain in each study were sought (e.g. for all measures, time points, analyses), and if not, the methods used to decide which results to collect.	Page 6-7-8
	10b	List and define all other variables for which data were sought (e.g. participant and intervention characteristics, funding sources). Describe any assumptions made about any missing or unclear information.	Page 7
Study risk of bias assessment	11	Specify the methods used to assess risk of bias in the included studies, including details of the tool(s) used, how many reviewers assessed each study and whether they worked independently, and if applicable, details of automation tools used in the process.	Page 7
Effect measures	12	Specify for each outcome the effect measure(s) (e.g. risk ratio, mean difference) used in the synthesis or presentation of results.	Page 8
Synthesis methods	13a	Describe the processes used to decide which studies were eligible for each synthesis (e.g. tabulating the study intervention characteristics and comparing against the planned groups for each synthesis (item #5)).	Page 7-8
	13b	Describe any methods required to prepare the data for presentation or synthesis, such as handling of missing summary statistics, or data conversions.	Page 8
	13c	Describe any methods used to tabulate or visually display results of individual studies and syntheses.	Page 8
	13d	Describe any methods used to synthesize results and provide a rationale for the choice(s). If meta-analysis was performed, describe the model(s), method(s) to identify the presence and extent of statistical heterogeneity, and software package(s) used.	Page 8
	13e	Describe any methods used to explore possible causes of heterogeneity among study results (e.g. subgroup analysis, meta-regression).	/
	13f	Describe any sensitivity analyses conducted to assess robustness of the synthesized results.	/
Reporting bias assessment	14	Describe any methods used to assess risk of bias due to missing results in a synthesis (arising from reporting biases).	Page 8
Certainty assessment	15	Describe any methods used to assess certainty (or confidence) in the body of evidence for an outcome.	/
**RESULTS**			
Study selection	16a	Describe the results of the search and selection process, from the number of records identified in the search to the number of studies included in the review, ideally using a flow diagram.	Page 9 and Figure 1
	16b	Cite studies that might appear to meet the inclusion criteria, but which were excluded, and explain why they were excluded.	Page 9
Study characteristics	17	Cite each included study and present its characteristics.	Table 1 and 2 and supplementary data
Risk of bias in studies	18	Present assessments of risk of bias for each included study.	Page 14 and figure 4
Results of individual studies	19	For all outcomes, present, for each study: (a) summary statistics for each group (where appropriate) and (b) an effect estimate and its precision (e.g. confidence/credible interval), ideally using structured tables or plots.	Page 13 and 14 and Table 3 and figure 2
Results of syntheses	20a	For each synthesis, briefly summarise the characteristics and risk of bias among contributing studies.	/
	20b	Present results of all statistical syntheses conducted. If meta-analysis was done, present for each the summary estimate and its precision (e.g. confidence/credible interval) and measures of statistical heterogeneity. If comparing groups, describe the direction of the effect.	Page 13 and 14 and table 3
	20c	Present results of all investigations of possible causes of heterogeneity among study results.	/
	20d	Present results of all sensitivity analyses conducted to assess the robustness of the synthesized results.	/
Reporting biases	21	Present assessments of risk of bias due to missing results (arising from reporting biases) for each synthesis assessed.	Page 14 and figure 3
Certainty of evidence	22	Present assessments of certainty (or confidence) in the body of evidence for each outcome assessed.	/
**DISCUSSION**			
Discussion	23a	Provide a general interpretation of the results in the context of other evidence.	Page 15-16-17
	23b	Discuss any limitations of the evidence included in the review.	Page 18-19
	23c	Discuss any limitations of the review processes used.	Page 18-19
	23d	Discuss implications of the results for practice, policy, and future research.	Page 18
**OTHER INFORMATION**			

Section and Topic	Item #	Checklist item	Location where item is reported
Registration and protocol	24a	Provide registration information for the review, including register name and registration number, or state that the review was not registered.	Page 24
	24b	Indicate where the review protocol can be accessed, or state that a protocol was not prepared.	Page 24
	24c	Describe and explain any amendments to information provided at registration or in the protocol.	/
Support	25	Describe sources of financial or non-financial support for the review, and the role of the funders or sponsors in the review.	Page 24
Competing interests	26	Declare any competing interests of review authors.	Page 24
Availability of data, code and other materials	27	Report which of the following are publicly available and where they can be found: template data collection forms; data extracted from included studies; data used for all analyses; analytic code; any other materials used in the review.	Page 20

From: Page MJ, McKenzie JE, Bossuyt PM, Boutron I, Hoffmann TC, Mulrow CD, et al. The PRISMA 2020 statement: an updated guideline for reporting systematic reviews. BMJ 2021;372:n71. doi: 10.1136/bmj.n71

For more information, visit: http://www.prisma-statement.org/

Included articles

1. Abel, D. *et al.* Encephalopathy and bilateral thalamic lesions in a child with MIS-C associated with COVID-19. *Neurology* **95**, 745-748, doi:10.1212/wnl.0000000000010652 (2020).
2. Akçay, N. *et al.* COVID-19-associated Acute Disseminated Encephalomyelitis-like Disease in 2 Children. *The Pediatric infectious disease journal* **40**, e445-e450, doi:10.1097/inf.0000000000003295 (2021).
3. Akhondian, J., Seilanian Toosi, F., Ashrafzadeh, F., Hashemi, N. & Saeedi Zand, N. J. I. C. N. J. COVID-19; neurological findings in five pediatric patients. **8**, 96-98 (2021).
4. Aksu Uzunhan, T. *et al.* Cytotoxic lesions of the corpus callosum in children: Etiology, clinical and radiological features, and prognosis. *Brain & development* **43**, 919-930, doi:10.1016/j.braindev.2021.05.001 (2021).
5. Al Haboob, A. A. Miller Fischer and posterior reversible encephalopathy syndromes post COVID-19 infection. *Neurosciences (Riyadh, Saudi Arabia)* **26**, 295-299, doi:10.17712/nsj.2021.3.20210002 (2021).
6. Aljomah, L. *et al.* Pediatrics COVID-19 and neurological manifestations: Single tertiary centre experience. *eNeurologicalSci* **24**, 100355, doi:10.1016/j.ensci.2021.100355 (2021).
7. Appavu, B. *et al.* Arteritis and Large Vessel Occlusive Strokes in Children After COVID-19 Infection. *Pediatrics* **147**, doi:10.1542/peds.2020-023440 (2021).
8. Asif, R. & MS, O. M. Rare complication of COVID-19 presenting as isolated headache. *BMJ case reports* **13**, doi:10.1136/bcr-2020-239275 (2020).
9. Balagurunathan, M., Natarajan, T., Karthikeyan, J., Palanisamy, V. J. C. & pediatrics, e. Clinical spectrum and short-term outcomes of multisystem inflammatory syndrome in children in a south Indian hospital. **64**, 531 (2021).
10. Bauer, S. C. *et al.* Pediatric COVID-19 delirium: case report of 2 adolescents. **120**, 131-136 (2021).
11. Becker, A. E., Chiotos, K., McGuire, J. L., Bruins, B. B. & Alcamo, A. M. Intracranial Hypertension in Multisystem Inflammatory Syndrome in Children. *The Journal of Pediatrics* **233**, 263-267, doi:https://doi.org/10.1016/j.jpeds.2021.02.062 (2021).
12. Bektaş, G., Akçay, N., Boydağ, K. & Şevketoğlu, E. Reversible splenial lesion syndrome associated with SARS-CoV-2 infection in two children. *Brain & development* **43**, 230-233, doi:10.1016/j.braindev.2020.10.002 (2021).
13. Bhatta, S., Sayed, A., Ranabhat, B., Bhatta, R. K. & Acharya, Y. New-Onset Seizure as the Only Presentation in a Child With COVID-19. *Cureus* **12**, e8820, doi:10.7759/cureus.8820 (2020).
14. Bhavsar, S. M. *et al.* COVID-19 Infection Associated With Encephalitis in an Adolescent. *Neurology. Clinical practice* **11**, e189-e192, doi:10.1212/cpj.0000000000000911 (2021).
15. Biglari, H. N., Sinaei, R., Pezeshki, S. & Hasani, F. K. J. I. j. o. c. n. Acute transverse myelitis of childhood due to novel coronavirus disease 2019: The first pediatric case report and review of literature. **15**, 107 (2021).
16. Biko, D. M. *et al.* Imaging of children with COVID-19: experience from a tertiary children's hospital in the United States. *Pediatric radiology* **51**, 239-247, doi:10.1007/s00247-020-04830-x (2021).
17. Brum, A. C. *et al.* Ischemic lesions in the brain of a neonate with SARS-CoV-2 infection. **40**, e340-e343 (2021).
18. Canham, L. J. W., Staniaszek, L. E., Mortimer, A. M., Nouri, L. F. & Kane, N. M. Electroencephalographic (EEG) features of encephalopathy in the setting of Covid-19: A case series. *Clinical neurophysiology practice* **5**, 199-205, doi:10.1016/j.cnp.2020.06.001 (2020).
19. Caro-Domínguez, P. *et al.* Imaging findings of multisystem inflammatory syndrome in children associated with COVID-19. *Pediatric radiology* **51**, 1608-1620, doi:10.1007/s00247-021-05065-0 (2021).
20. Cecchini, M. P. *et al.* Persistent chemosensory dysfunction in a young patient with mild COVID-19 with partial recovery 15 months after the onset. *Neurological sciences : official journal of the Italian Neurological Society and of the Italian Society of Clinical Neurophysiology* **43**, 99-104, doi:10.1007/s10072-021-05635-y (2022).
21. Chen, L. J. F. N. Association of acute disseminated encephalomyelitis (ADEM) and COVID-19 in a pediatric patient. **2**, 19-19 (2021).
22. Chiotos, K. *et al.* Multisystem Inflammatory Syndrome in Children During the Coronavirus 2019 Pandemic: A Case Series. *Journal of the Pediatric Infectious Diseases Society* **9**, 393-398, doi:10.1093/jpids/piaa069 (2020).
23. Coronado Munoz, A. *et al.* High incidence of stroke and mortality in pediatric critical care patients with COVID-19 in Peru. *Pediatric research* **91**, 1730-1734, doi:10.1038/s41390-021-01547-x (2022).
24. Curtis, M. *et al.* Guillain-Barré Syndrome in a Child With COVID-19 Infection. *Pediatrics* **147**, doi:10.1542/peds.2020-015115 (2021).
25. de Miranda Henriques-Souza, A. M. *et al.* Acute disseminated encephalomyelitis in a COVID-19 pediatric patient. *Neuroradiology* **63**, 141-145, doi:10.1007/s00234-020-02571-0 (2021).
26. de Oliveira, M. R., Lucena, A. R. V. P., Higino, T. M. M. & Ventura, C. V. Oculomotor nerve palsy in an asymptomatic child with COVID-19. *Journal of American Association for Pediatric Ophthalmology and Strabismus* **25**, 169-170, doi:https://doi.org/10.1016/j.jaapos.2021.02.001 (2021).
27. Dean, A., Said, A., Marri, K. & Chelius, D. Stridor Due to Cranial Nerve X Palsy Progressing to Polyneuropathy in a Teenager With COVID-19. *Pediatrics* **148**, doi:10.1542/peds.2021-051534 (2021).
28. Divya, K., Indumathi, C., Vikrant, K. & Padmanaban, S. Pseudotumor Cerebri Complicating Multisystem Inflammatory Syndrome in a Child. *Journal of current ophthalmology* **33**, 358-362, doi:10.4103/joco.joco_241_20 (2021).
29. Dugue, R. *et al.* Neurologic manifestations in an infant with COVID-19. *Neurology* **94**, 1100-1102, doi:10.1212/wnl.0000000000009653 (2020).
30. Elmas, B. *et al.* Evaluation of taste and smell disorders in pediatric COVID-19 Cases. *Revista da Associacao Medica Brasileira (1992)* **67**, 789-794, doi:10.1590/1806-9282.20200547 (2021).
31. Emami, A. *et al.* Seizure in patients with COVID-19. *Neurological sciences : official journal of the Italian Neurological Society and of the Italian Society of Clinical Neurophysiology* **41**, 3057-3061, doi:10.1007/s10072-020-04731-9 (2020).
32. Farley, M. & Zuberi, J. COVID-19 Precipitating Status Epilepticus in a Pediatric Patient. *The American journal of case reports* **21**, e925776, doi:10.12659/ajcr.925776 (2020).
33. Fenlon Iii, E. P., Chen, S., Ruzal-Shapiro, C. B., Jaramillo, D. & Maddocks, A. B. R. Extracardiac imaging findings in COVID-19-associated multisystem inflammatory syndrome in children. *Pediatric radiology* **51**, 831-839, doi:10.1007/s00247-020-04929-1 (2021).
34. Foster, C. H., Vargas, A. J., Wells, E., Keating, R. F. & Magge, S. N. J. J. o. N. C. L. Cerebral vasculopathy and strokes in a child with COVID-19 antibodies: illustrative case. **2** (2021).

35. Fouriki, A. *et al.* Case Report: Case Series of Children With Multisystem Inflammatory Syndrome Following SARS-CoV-2 Infection in Switzerland. *Frontiers in pediatrics* **8**, 594127, doi:10.3389/fped.2020.594127 (2020).
36. Fragoso, D. C. *et al.* COVID-19 as a cause of acute neonatal encephalitis and cerebral cytotoxic edema. **40**, e270-e271 (2021).
37. Frank, C. H. M. *et al.* Guillain–Barré Syndrome Associated with SARS-CoV-2 Infection in a Pediatric Patient. *Journal of Tropical Pediatrics* **67**, fmaa044, doi:10.1093/tropej/fmaa044 (2021).
38. García-Howard, M. *et al.* Case Report: Benign Infantile Seizures Temporally Associated With COVID-19. *Frontiers in pediatrics* **8**, 507, doi:10.3389/fped.2020.00507 (2020).
39. Gaughan, M. *et al.* Pediatric Parainfectious Encephalitis Associated With COVID-19. *Neurology* **96**, 541-544, doi:10.1212/wnl.0000000000011476 (2021).
40. Gaur, P., Dixon, L., Jones, B., Lyall, H. & Jan, W. COVID-19-Associated Cytotoxic Lesions of the Corpus Callosum. *AJNR. American journal of neuroradiology* **41**, 1905-1907, doi:10.3174/ajnr.A6713 (2020).
41. Giannantonio, S., Scorpecci, A., Montemurri, B. & Marsella, P. Case of COVID-19-induced vestibular neuritis in a child. *BMJ case reports* **14**, doi:10.1136/bcr-2021-242978 (2021).
42. Gulko, E. *et al.* Vessel Wall Enhancement and Focal Cerebral Arteriopathy in a Pediatric Patient with Acute Infarct and COVID-19 Infection. *AJNR. American journal of neuroradiology* **41**, 2348-2350, doi:10.3174/ajnr.A6778 (2020).
43. Gupta Dch, S. *et al.* Unusual Clinical Manifestations and Outcome of Multisystem Inflammatory Syndrome in Children (MIS-C) in a Tertiary Care Hospital of North India. *Journal of tropical pediatrics* **67**, doi:10.1093/tropej/fmaa127 (2021).
44. Hameed, S. *et al.* Spectrum of Imaging Findings at Chest Radiography, US, CT, and MRI in Multisystem Inflammatory Syndrome in Children Associated with COVID-19. *Radiology* **298**, E1-e10, doi:10.1148/radiol.2020202543 (2021).
45. Hatipoglu, N., Yazici, Z. M., Palabiyik, F., Gulustan, F. & Sayin, I. Olfactory bulb magnetic resonance imaging in SARS-CoV-2-induced anosmia in pediatric cases. *International journal of pediatric otorhinolaryngology* **139**, 110469, doi:10.1016/j.ijporl.2020.110469 (2020).
46. Hossain, M. *et al.* COVID-19: A NEW THREAT TO THE NERVOUS SYSTEM IN CHILDREN.
47. Hosseinpour, S. J. C. R. i. M. S. Suspected case of COVID-19-associated Guillain-Barre Syndrome in an Iranian child. **5**, 52-57 (2021).
48. Hutchison, L., Plichta, A. M., Lerea, Y., Madora, M. & Ushay, H. M. Neuropsychiatric Symptoms in an Adolescent Boy With Multisystem Inflammatory Syndrome in Children. *Psychosomatics* **61**, 739-744, doi:https://doi.org/10.1016/j.psym.2020.06.015 (2020).
49. Insuga, V. S. *et al.* Pseudotumor cerebri caused by SARS-CoV-2 infection in a boy. **19**, 207-209 (2021).
50. Jillella, D. V. *et al.* Successful Endovascular Therapy in COVID-19 Associated Pediatric Ischemic Stroke. *Journal of Stroke and Cerebrovascular Diseases* **30**, 106152, doi:https://doi.org/10.1016/j.jstrokecerebrovasdis.2021.106152 (2021).
51. Khair, A. Intermittent Frontal Rhythmic Discharges as an Electroencephalogram Biomarker of Acute SARS-CoV-2 Infection-Associated Encephalopathy in Children. *Cureus* **13**, e19149, doi:10.7759/cureus.19149 (2021).
52. Khalifa, M. *et al.* Guillain-Barré Syndrome Associated With Severe Acute Respiratory Syndrome Coronavirus 2 Detection and Coronavirus Disease 2019 in a Child. *Journal of the Pediatric Infectious Diseases Society* **9**, 510-513, doi:10.1093/jpids/piaa086 (2020).
53. Khan, A. *et al.* Clinical spectrum of neurological manifestations in pediatric COVID-19 illness: a case series. **67**, fmab059 (2021).
54. Khera, D., Didel, S., Panda, S., Tiwari, S. & Singh, K. J. T. P. I. D. J. Concurrent longitudinally extensive transverse myelitis and Guillain-Barré syndrome in a child secondary to COVID-19 infection: a severe neuroimmunologic complication of COVID-19. **40**, e236-e239 (2021).
55. Khoshnood, M., Mahabir, R., Shillingford, N. M. & Santoro, J. D. Post-infectious inflammatory syndrome associated with SARS-CoV-2 in a paediatric patient with Down syndrome. *BMJ case reports* **14**, doi:10.1136/bcr-2020-240490 (2021).
56. Khosravi, B., Moradveisi, B., Abedini, M., Behzadi, S. & Karimi, A. Stroke in a child with SARS-CoV-2 infection: A case report. *eNeurologicalSci* **23**, 100345, doi:https://doi.org/10.1016/j.ensci.2021.100345 (2021).
57. Korkmazer, B. *et al.* Posterior reversible encephalopathy syndrome in a pediatric COVID-19 patient. **40**, e240-e242 (2021).
58. Krueger, M. B. *et al.* A wide spectrum of neurological manifestations in pediatrics patients with the COVID-19 infection: a case series. **27**, 782-786 (2021).
59. Kushwaha, S. *et al.* Neurological Associations of COVID-19-Do We Know Enough: A Tertiary Care Hospital Based Study. *Frontiers in neurology* **11**, 588879, doi:10.3389/fneur.2020.588879 (2020).
60. Lazarte-Rantes, C., Guevara-Castañón, J., Romero, L. & Guillén-Pinto, D. Acute Necrotizing Encephalopathy Associated With SARS-CoV-2 Exposure in a Pediatric Patient. *Cureus* **13**, e15018, doi:10.7759/cureus.15018 (2021).
61. Lindan, C. E. *et al.* Neuroimaging manifestations in children with SARS-CoV-2 infection: a multinational, multicentre collaborative study. *The Lancet. Child & adolescent health* **5**, 167-177, doi:10.1016/s2352-4642(20)30362-x (2021).
62. McLendon, L. A., Rao, C. K., Da Hora, C. C., Islamovic, F. & Galan, F. N. Post-COVID-19 Acute Disseminated Encephalomyelitis in a 17-Month-Old. *Pediatrics* **147**, doi:10.1542/peds.2020-049678 (2021).
63. Mehra, B. *et al.* COVID-19-associated severe multisystem inflammatory syndrome in children with encephalopathy and neuropathy in an adolescent girl with the successful outcome: an unusual presentation. **24**, 1276 (2020).
64. Miller, E. H. *et al.* in *Open forum infectious diseases.* ofaa501 (Oxford University Press US).
65. Ngo, B. *et al.* Cerebrospinal fluid cytokine, chemokine, and SARS-CoV-2 antibody profiles in children with neuropsychiatric symptoms associated with COVID-19. *Multiple Sclerosis and Related Disorders* **55**, 103169, doi:https://doi.org/10.1016/j.msard.2021.103169 (2021).
66. Olivotto, S. *et al.* Acute encephalitis in pediatric multisystem inflammatory syndrome associated with COVID-19. *European journal of paediatric neurology : EJPN : official journal of the European Paediatric Neurology Society* **34**, 84-90, doi:10.1016/j.ejpn.2021.07.010 (2021).
67. Orman, G. *et al.* Neuroimaging Offers Low Yield in Children Positive for SARS-CoV-2. *AJNR. American journal of neuroradiology* **42**, 951-954, doi:10.3174/ajnr.A7022 (2021).
68. Oualha, M. *et al.* Severe and fatal forms of COVID-19 in children. *Archives de pediatrie : organe officiel de la Societe francaise de pediatrie* **27**, 235-238, doi:10.1016/j.arcped.2020.05.010 (2020).
69. Palabiyik, F. *et al.* Imaging of Multisystem Inflammatory Disease in Children (MIS-C) Associated With COVID-19. *Academic radiology* **28**, 1200-1208, doi:10.1016/j.acra.2021.05.030 (2021).

70. Paterson, R. W. *et al.* The emerging spectrum of COVID-19 neurology: clinical, radiological and laboratory findings. *Brain : a journal of neurology* **143**, 3104-3120, doi:10.1093/brain/awaa240 (2020).
71. Penner, J. *et al.* 6-month multidisciplinary follow-up and outcomes of patients with paediatric inflammatory multisystem syndrome (PIMS-TS) at a UK tertiary paediatric hospital: a retrospective cohort study. *The Lancet Child & Adolescent Health* **5**, 473-482, doi:https://doi.org/10.1016/S2352-4642(21)00138-3 (2021).
72. Powers, K. T. & Santoro, J. D. Metabolic stroke-like episode in a child with FARS2 mutation and SARS-CoV-2 positive cerebrospinal fluid. *Molecular Genetics and Metabolism Reports* **27**, 100756, doi:https://doi.org/10.1016/j.ymgmr.2021.100756 (2021).
73. Ray, S. T. J. *et al.* Neurological manifestations of SARS-CoV-2 infection in hospitalised children and adolescents in the UK: a prospective national cohort study. *The Lancet Child & Adolescent Health* **5**, 631-641, doi:https://doi.org/10.1016/S2352-4642(21)00193-0 (2021).
74. Riollano-Cruz, M. *et al.* Multisystem inflammatory syndrome in children related to COVID-19: A New York City experience. *Journal of medical virology* **93**, 424-433, doi:10.1002/jmv.26224 (2021).
75. Saeed, A. & Shorafa, E. Status epilepticus as a first presentation of COVID-19 infection in a 3 years old boy; Case report and review the literature. *IDCases* **22**, e00942, doi:10.1016/j.idcr.2020.e00942 (2020).
76. Sahu, P. J. I. J. o. C. H. Acute disseminated encephalomyelitis in a SARS-CoV2 seropositive child. 249-251 (2021).
77. Salman, H. *et al.* COVID-19-associated multisystem inflammatory syndrome in children: Experiences of three centres in Turkey. **32**, 460-466 (2022).
78. Sánchez-Morales, A. E. *et al.* Neurological manifestations temporally associated with SARS-CoV-2 infection in pediatric patients in Mexico. **37**, 2305-2312 (2021).
79. Sandoval, F. *et al.* Neurologic Features Associated With SARS-CoV-2 Infection in Children: A Case Series Report. *Journal of child neurology* **36**, 853-866, doi:10.1177/0883073821989164 (2021).
80. Sarigecili, E., Arslan, I., Ucar, H. K. & Celik, U. Pediatric anti-NMDA receptor encephalitis associated with COVID-19. *Child's nervous system : ChNS : official journal of the International Society for Pediatric Neurosurgery* **37**, 3919-3922, doi:10.1007/s00381-021-05155-2 (2021).
81. Schiff, J. & Brennan, C. Covid-19 presenting as a bulging fontanelle. *The American Journal of Emergency Medicine* **43**, 81-82, doi:https://doi.org/10.1016/j.ajem.2021.01.062 (2021).
82. Shala, N. *et al.* Case Report A 14-Year-Old Male Patient with Kawasaki Disease Presented with Stroke after COVID-19. (2021).
83. Shenker, J., Trogen, B., Schroeder, L., Ratner, A. J. & Kahn, P. Multisystem Inflammatory Syndrome in Children Associated with Status Epilepticus. *The Journal of pediatrics* **227**, 300-301, doi:10.1016/j.jpeds.2020.07.062 (2020).
84. Sofijanova, A. *et al.* Severe Encephalitis in Infant with COVID-19: A Case Report. *Open Access Macedonian Journal of Medical Sciences* **8**, 514-517, doi:10.3889/oamjms.2020.5485 (2020).
85. Sofuoğlu, A., Akçay, N., Şevketoğlu, E. & Bektaş, G. Pseudotumor Cerebri Syndrome as a Neurologic Involvement of Multisystem Inflammatory Syndrome in Children: A Case Report. *Journal of tropical pediatrics* **67**, doi:10.1093/tropej/fmab075 (2021).
86. Tiwari, L., Shekhar, S., Bansal, A. & Kumar, S. COVID-19 associated arterial ischaemic stroke and multisystem inflammatory syndrome in children: a case report. *The Lancet. Child & adolescent health* **5**, 88-90, doi:10.1016/s2352-4642(20)30314-x (2021).
87. Tomar, L. R., Shah, D. J., Agarwal, U., Batra, A. & Anand, I. J. M. D. C. P. Acute post-infectious cerebellar ataxia due to covid-19. **8**, 610 (2021).
88. Ucan, B. *et al.* Multisystem inflammatory syndrome in children associated with SARS-CoV-2: extracardiac radiological findings. *The British journal of radiology* **95**, 20210570, doi:10.1259/bjr.20210570 (2022).
89. Urso, L. *et al.* The case of encephalitis in a COVID-19 pediatric patient. *Neurological sciences : official journal of the Italian Neurological Society and of the Italian Society of Clinical Neurophysiology* **43**, 105-112, doi:10.1007/s10072-021-05670-9 (2022).
90. Vivanti, A. J. *et al.* Transplacental transmission of SARS-CoV-2 infection. **11**, 1-7 (2020).
91. Vraka, K. *et al.* Two Paediatric Patients with Encephalopathy and Concurrent COVID-19 Infection: Two Sides of the Same Coin? *Case reports in neurological medicine* **2021**, 6658000, doi:10.1155/2021/6658000 (2021).
92. Wang, E., Ulualp, S. O., Liu, C. & Veling, M. Sudden anosmia and ageusia in a child: A COVID-19 case report. *Otolaryngology Case Reports* **18**, 100267, doi:https://doi.org/10.1016/j.xocr.2021.100267 (2021).
93. Yan, K. *et al.* Effects of SARS-CoV-2 infection on neuroimaging and neurobehavior in neonates. **17**, 171-179 (2021).
94. Yildiz, H. *et al.* COVID-19-associated cerebral white matter injury in a newborn infant with afebrile seizure. **40**, e268-e269 (2021).
95. Zain, S., Petropoulou, K., Mirchia, K., Hussien, A. & Mirchia, K. COVID-19 as a rare cause of facial nerve neuritis in a pediatric patient. *Radiology case reports* **16**, 1400-1404, doi:10.1016/j.radcr.2021.03.063 (2021).
96. Zubarioglu, T. *et al.* COVID-19 triggered encephalopathic crisis in a patient with glutaric aciduria type 1. **34**, 1611-1614 (2021).
